# Site-1 protease ablation in the osterix-lineage in mice results in bone marrow neutrophilia and hematopoietic stem cell alterations

**DOI:** 10.1242/bio.052993

**Published:** 2020-06-23

**Authors:** Debabrata Patra, Joongho Kim, Qiang Zhang, Eric Tycksen, Linda J. Sandell

**Affiliations:** 1 Department of Orthopaedic Surgery, Washington University School of Medicine, St. Louis, MO 63110, USA; 2 Department of Medicine, Washington University School of Medicine, St. Louis, MO 63110, USA; 3 McDonnell Genome Institute, Department of Genetics, Washington University School of Medicine, St. Louis, MO 63110, USA

**Keywords:** Site-1 protease, Stefin, Osterix, Neutrophilia, Hematopoietic stem cells, Osteopenia

## Abstract

Site-1 protease (S1P) ablation in the osterix-lineage in mice drastically reduces bone development and downregulates bone marrow-derived skeletal stem cells. Here we show that these mice also suffer from spina bifida occulta with a characteristic lack of bone fusion in the posterior neural arches. Molecular analysis of bone marrow-derived non-red blood cell cells, via single-cell RNA-Seq and protein mass spectrometry, demonstrate that these mice have a much-altered bone marrow with a significant increase in neutrophils and *Ly6C*-expressing leukocytes. The molecular composition of bone marrow neutrophils is also different as they express more and additional members of the stefin A (Stfa) family of proteins. *In vitro*, recombinant Stfa1 and Stfa2 proteins have the ability to drastically inhibit osteogenic differentiation of bone marrow stromal cells, with no effect on adipogenic differentiation. FACS analysis of hematopoietic stem cells show that despite a decrease in hematopoietic stem cells, S1P ablation results in an increased production of granulocyte-macrophage progenitors, the precursors to neutrophils. These observations indicate that S1P has a role in the lineage specification of hematopoietic stem cells and/or their progenitors for development of a normal hematopoietic niche. Our study designates a fundamental requirement of S1P for maintaining a balanced regenerative capacity of the bone marrow niche.

## Abstract (precis)

**Summary:** Ablation of S1P in the osterix-lineage downregulates bone development due to hematopoietic progenitor alterations that increase bone marrow neutrophils with accompanying increases in stefin proteins with osteogenic inhibitory activity.

## INTRODUCTION

Site-1 protease (S1P), coded by the *Mbtps1* gene (*membrane bound transcription factor protease, site 1*), is a proprotein convertase known primarily for its vital roles in lipid homeostasis and the unfolded protein response (UPR) (Brown et al., 2000; Eberlé et al., 2004). These pathways are fundamental to cellular homeostasis and involve the activation of latent, ER-membrane-bound transcription factors, although non-transcription factor substrates such as *N*-acetylglucosamine-1-phosphotransferase α/β-subunit precursor in lysosomal biogenesis have been reported (Marschner et al., 2011). In previous studies, we have demonstrated the importance of S1P to overall skeletal development in mice. The ablation of S1P in chondroprogenitors resulted in mice with no endochondral bone development (Patra et al., 2007), but its ablation in postnatal chondrocytes prevented chondrocyte maturation in the postnatal growth plate followed by a complete elimination of the primary growth plate (Patra et al., 2011), resulting in shortened bones and a profound effect on trabecular bone growth. These investigations demonstrated that the major cell type affected was chondrocytes, which were unable to secrete type II collagen followed by chondrocyte apoptosis; these events may have led to a secondary effect on both growth and development.

Our studies to analyze a direct role for S1P in bone development by ablating S1P in the osterix (Osx)-lineage demonstrated a direct role in bone development with minimal effect on chondrocyte maturation or type II collagen secretion (Patra et al., 2018). These mice had osteopenia with significant reductions in bone formation and mineral apposition rates with drastically reduced skeletal stem cells (SSCs) in the bone marrow. Besides having delicate bones that fractured easily, these mice also developed cervical and thoracic scoliosis to varying degrees very early postnatally. Furthermore, bone marrow stromal cells, which showed normal adipogenic differentiation, were unable to differentiate into osteoblasts *in vitro*.

In this study, we have analyzed mice with S1P ablation in the Osx-lineage in greater detail. Specifically, we investigated the scoliotic spines of these mice further and have studied their bone marrow compartment extensively by using several next generation sequencing technologies such as single cell sequencing of bone marrow cells, and proteomic analysis of proteins derived from total bone marrow cells [minus red blood cells (RBC)]. Besides scoliosis, these mice also have spina bifida occulta (SBO). Our studies show that S1P ablation in the Osx-lineage results in bone marrow neutrophilia with extensive inflammation-like condition of the bone marrow compartment. The hematopoietic environment in the bone marrow of these mice is drastically different from the wild type (WT) with two different populations of neutrophils, a population that is similar to WT and a population that overexpresses additional members of the stefin A (Stfa) family of proteins. *In vitro*, recombinant Stfa proteins have the ability to selectively hinder osteogenic differentiation. Extensive characterization of hematopoietic stem cells revealed that despite a decrease in long-term, multipotent LIN^−^ Sca1^+^ c-Kit^+^ (LSK) and LSK signaling lymphocytic activation molecule (SLAM) hematopoietic progenitors, these mice exhibit a significant increase in granulocyte-macrophage progenitors (GMP), the precursors to neutrophils and other leukocytes.

## RESULTS

### SBO in mice with S1P ablation in the osterix lineage

**Fig. 1.**
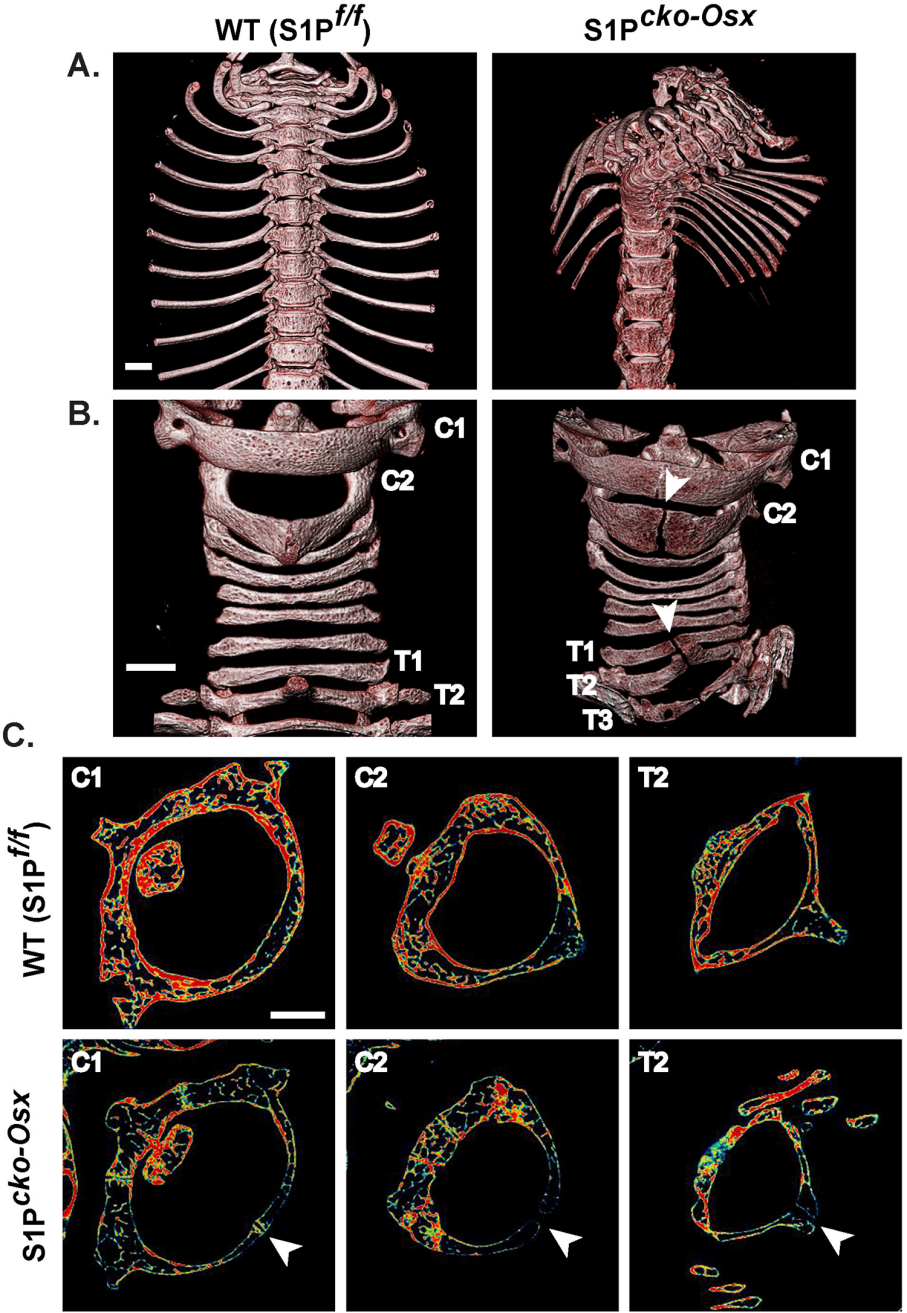
**Spina bifida occulta (SBO) in addition to scoliosis in S1P*^cko−Osx^* (Cko) mice.** µCT images of the cervical and thoracic spine showing scoliosis (A) and SBO (B) (arrowheads) in the C2 (Axis), T1, T2 and T3 vertebrae in Cko mice when compared to the WT (S1P*^f/f^*) mice. Images are shown in eight-color realistic color scheme. (C) Images of individual scan slides from µCT (in jet color scheme) through the center of C1 (Atlas), C2 (Axis) and T2 vertebrae showing the lack of fusion (arrowheads) of the spinous processes in the posterior arches of the vertebrae in the Cko. Representative images are shown from studies of several different litters (*N*=6). Scale bars: 1 mm.

**Fig. 2.**
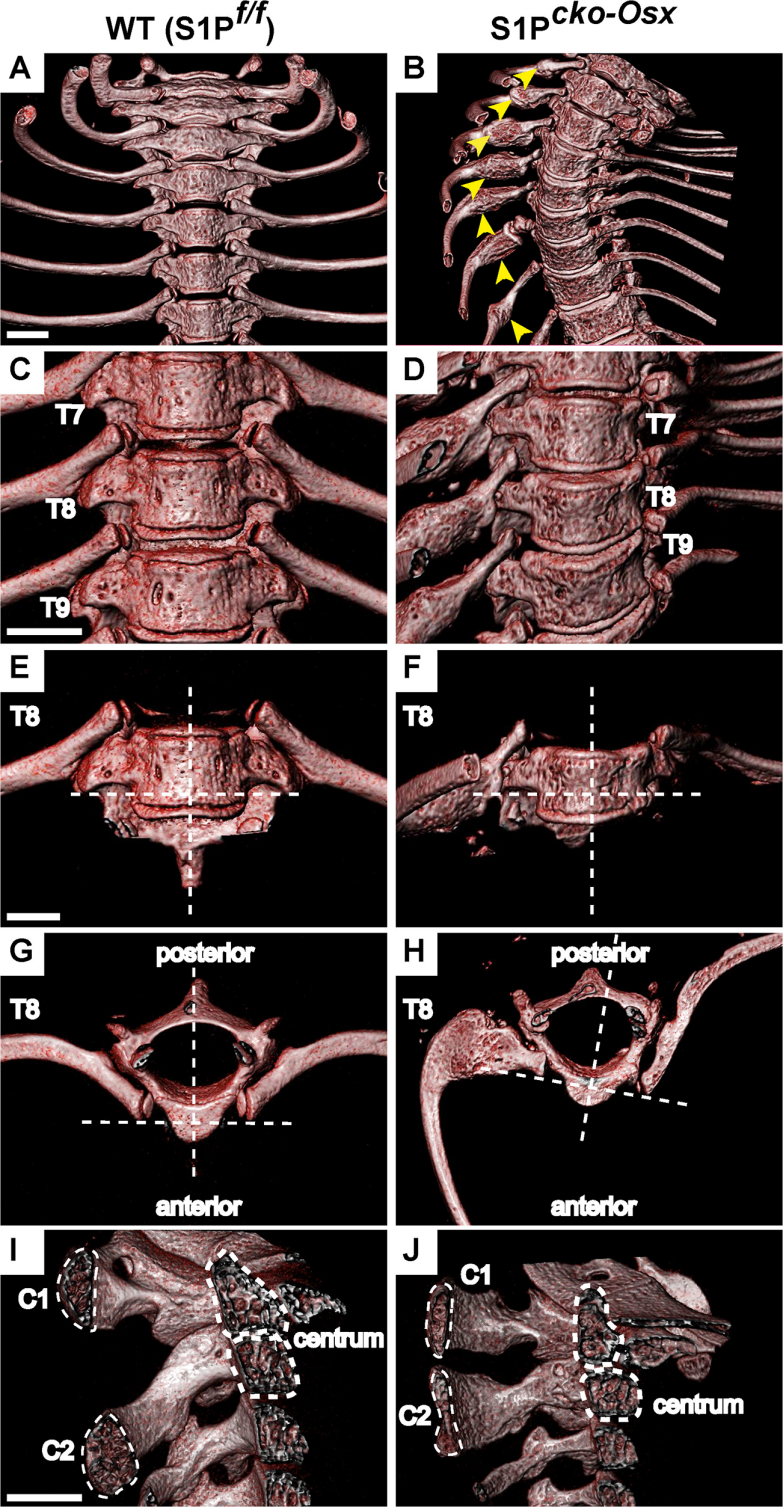
**Deviations from normal in the S1P*^cko-Osx^* spine.** (A,B) µCT images (using 8 Color Realistic 3D lookup table) showing hyperplastic fractures (yellow arrows) in the ribs of the Cko, absent in the WT. The anterior side (frontal view) of the spine is shown. (C,D) Disproportionate vertebrae development with scoliotic twist as exemplified by T7-T9 thoracic vertebrae (anterior side shown), and shown in detail for T8 (E–H). The cross bars in E–H highlight the lack of symmetry in the Cko, made profound by the scoliosis. Notice the uneven thickness of the sides of T8 at the site of rib attachment to the vertebrae (E,F) and the asymmetrical development of the neural tube and the positioning of the ribs in T8 (G,H). (I,J) µCT images through the center of the centrum (outlined) of C1 and C2 vertebrae and their corresponding sinuous processes (outlined). Scale bars: 1 mm.

We previously reported that mice with S1P ablation in the Osx-lineage, i.e. S1P*^cko-Osx^* (Cko) mice show scoliosis to varying degrees, often severe that develops very early postnatally (Patra et al., 2018). We investigated the physical characteristics of these scoliotic spines further using computed tomography (µCT) and discovered that in addition to scoliosis (Fig. 1A) Cko mice also have SBO, a malformation in the vertebrae caused due to the lack of fusion of the spinous processes (neural arches) of the vertebrae on the posterior side (Fig. 1B). SBO was most commonly seen in the cervical C1 (Atlas) and C2 (Axis) and the thoracic T1 and T2 vertebral elements of the Cko spine (Fig. 1B,C). Besides scoliosis and SBO, Cko mice almost invariably suffered from hyperplastic fracture of the ribs on the right side of the spine (Fig. 2A,B) and asymmetric development of the vertebrae within the scoliotic portion of the spine (Fig. 2C–H) where the left side of the vertebrae, in the same direction as the bend of the scoliosis, is thinner than the right side. The twist from the scoliosis also induces an abnormal shape of the rib cage (Fig. 2B) with abnormal attachment of the ribs to the vertebrae as shown for T8 (Fig. 2H). In general, as true for the limbs in these mice (Patra et al., 2018), the vertebral elements are also smaller in the Cko as exemplified by cross sections of the centrums in C1 and C2 (Fig. 2I,J).

**Fig. 3.**
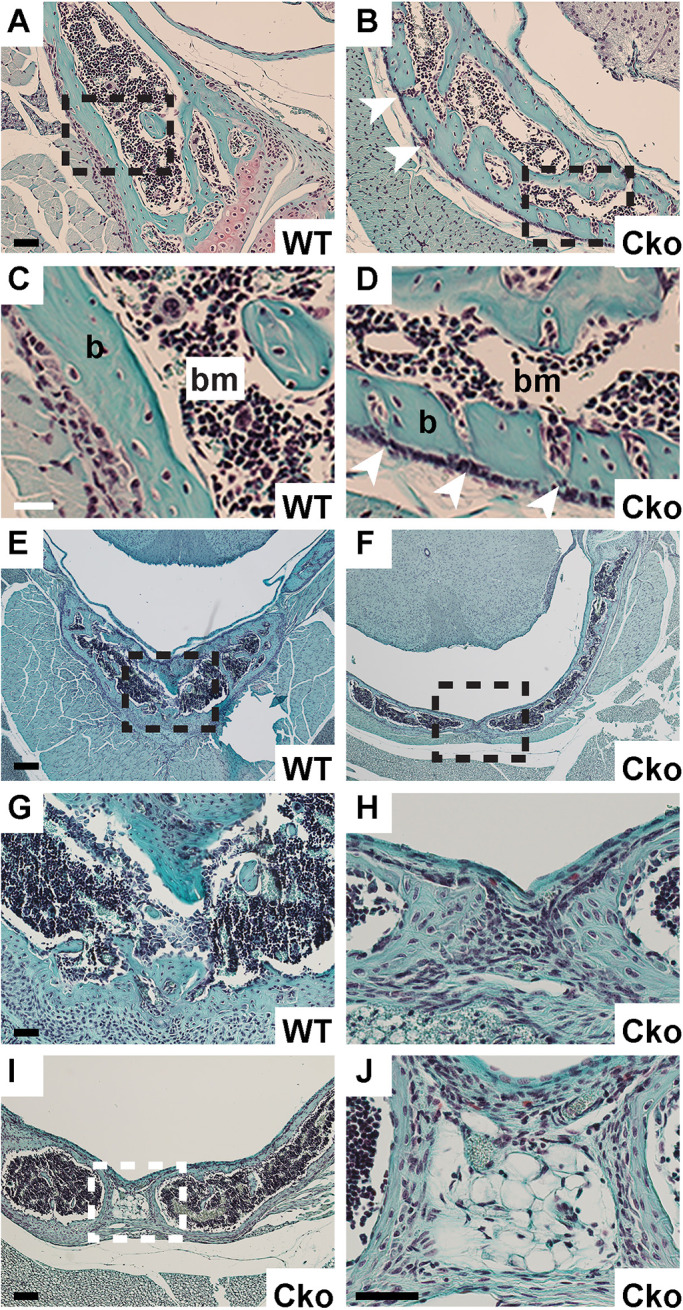
**Histological analysis of SBO.** (A–D) Safranin O stained cross-sections of C2 vertebrae showing the histology of the bony portion of the posterior arch in WT and Cko. C and D are higher magnification images of the outlined regions in A and B, respectively. Arrowheads in B and D highlight fissures in the bone, suggesting leakage of the bone marrow (b, bone; bm, bone marrow). (E–J) Safranin O stained sections of the tip of the neural arch showing lack of fusion of the spinous processes in the Cko resulting in SBO. G and H are higher magnification images of the outlined regions in E and F, respectively. Panel I shows a different perspective of SBO in the Cko C2 at a different depth of the vertebrae. Panel J is a higher magnification image of the outlined region in I. Scale bars: (A,B): 50 µm; (C,D): 25 µm; (E,F): 200 µm; (G,H): 50 µm; (I) 100 µm; (J) 50 µm.

To understand the nature of the SBO, we conducted a histological analysis of the C2 vertebrae in the WT and Cko (Fig. 3). Interestingly, there was more than one abnormality in the Cko C2 vertebrae. In the WT, the bone in the posterior arch of the vertebrae (that continues to form the spinous process) are continuous and solid with the bone marrow contained within the bone marrow cavity. In the Cko, however, the bones have fissures with bone marrow appearing to leak from the bone marrow compartment to the outside (Fig. 3A–D). Histological analyses revealed that the bones of the spinous process did not grow completely to fuse at the tips of the posterior arch. This lack of ossification at this site results in the typical feature of the gap seen in the spinous process in SBO (Fig. 3E–J). The tissue between the tips of the bones in the spinous process do not resemble cartilage and appear to be unossified tissue (Fig. 3H,J) of undetermined lineage. The absence of cartilage tissue between the two unfused tips in SBO in the Cko was confirmed by immunofluorescence staining for type II collagen (a marker for cartilage tissue) (Fig. S1). Thus, the inability to undergo osteogenesis due to the lack of S1P in the Osx-lineage as reported in our earlier study, affects vertebrae development drastically as well. The hyperplastic fractures, fissures in the vertebral bones and SBO are all outcomes of the global downregulation of bone development in these mice.

### Upregulation of the stefin family of proteins in the Cko bone marrow

**Fig. 4.**
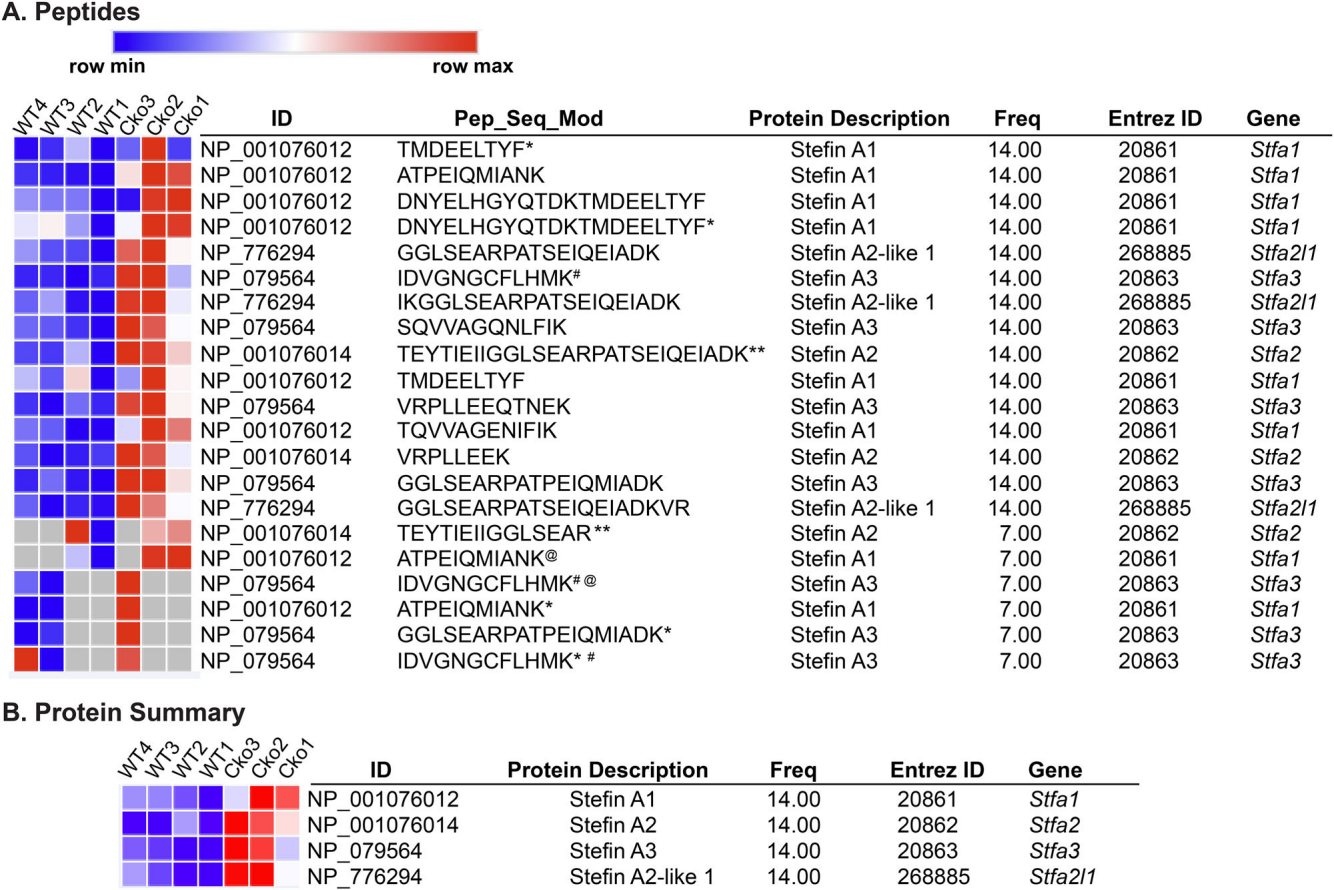
**A Morpheus heatmap (https://software.broadinstitute.org/morpheus) of the Stfa peptides (A) and proteins (B) identified by LC/MS/MS showing their overexpression in Cko BMCs (*N*=3), when compared to the WT (*N*=4) (also see Fig. S2 and** **Table 1).** The peptide sequences along with any modifications identified are shown (Pep-Seq-Mod). Modified forms of peptides identified are delineated as follows: *: Oxidation; #: Carbamidomethyl; **: Acetyl (Protein N-term); @: Deamidated.

Bone marrow stromal cells isolated from Cko mice were unable to demonstrate osteogenesis *in vitro* and demonstrated a significant reduction in SSCs (Patra et al., 2018). To investigate a molecular connection between the bone, bone marrow, spinal aberrations and SSC downregulation, we investigated RBC-free bone marrow cells by a number of different approaches. We harvested non-RBC bone marrow cells (BMCs) from WT and Cko mice, lysed them and performed differential proteomics on the protein lysate to analyze how the bone marrow proteome in the Cko differed from the WT due to the absence of S1P in the Osx-lineage. Proteomic analysis was performed using tryptic digestion and LC/MS/MS analysis after tandem mass tag (TMT) labeling. A preliminary study performed with BMCs harvested from one WT, Het and Cko mice demonstrated that the Stfa family of proteins was significantly upregulated in BMC lysates from Cko mice, with the WT and Het showing similar profiles (Fig. S2). Further studies were therefore restricted to WT and Cko mice only. Table 1 shows the top 25 peptide sequences upregulated in the Cko identified by LC/MS/MS; Table 2 shows the top 15 proteins identified as upregulated, derived from the peptides identified in Table 1. The stefin proteins are part of a gene cluster of highly homologous proteins that include *Stfa1*, *Stfa2*, *Stfa2l1* (*stefin A2-like 1*), *Stfa3*, *Gm5483*, *Gm5416*, *BC100530*, *BC117090*, *Csta* and the *2010005H15Rik* genes, present on mouse chromosome 16 (Rhead et al., 2010). Fig. 4A shows a graphical representation, using Morpheus, of the different peptides belonging to Stfa1, Stfa2, Stfa3 and Stfa2l1 proteins along with their relative distribution in WT and Cko mice. Fig. 4B shows a graphical summary of the relative protein distribution for Stfa1/Stfa2/Stfa3/Stfa2l1 for WT and Cko. These data clearly demonstrate that Stfa1, Stfa2, Stfa2l1, Stfa3, Gm5483 and BC100530 proteins are statistically significantly upregulated in Cko BMCs. Stefins are cytoplasmic inhibitors of proteases such as cathepsins (Turk et al., 2012), and are part of the normal expression profile of bone marrow/hematopoietic tissue (Bilodeau et al., 2009). Their upregulation in the bone marrow therefore suggests a disruption of the hematopoietic niche in Cko mice. Pathway analysis with upregulated proteins using Gene Ontology (GO) (Table S1) also identified several pathways related to the hematopoietic system in general that were upregulated (such as those related to Cxcr chemokine activity) or disrupted (negative regulation of MHC class II, negative regulation of megakaryocyte differentiation, negative regulation of hematopoietic progenitor cell differentiation) in the Cko. This analysis also identified proteins that were downregulated in Cko BMCs (Table S2). But given the shared identification of stefins as upregulated between proteomics and RNA-Seq analysis (see below), these were not pursued further.

**Table 1.**
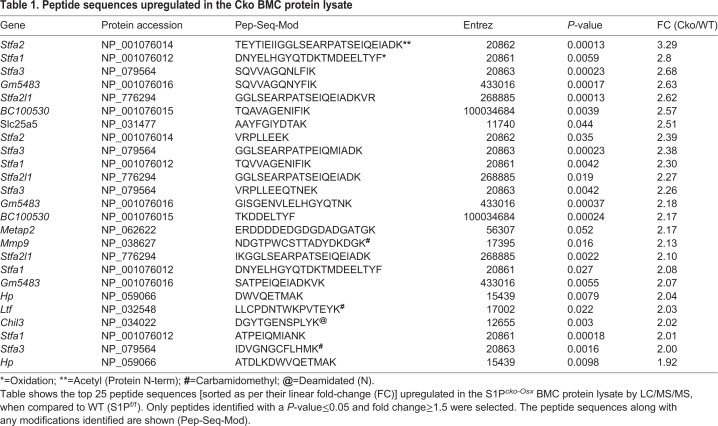
**Peptide sequences upregulated in the Cko BMC protein lysate**

**Table 2.**
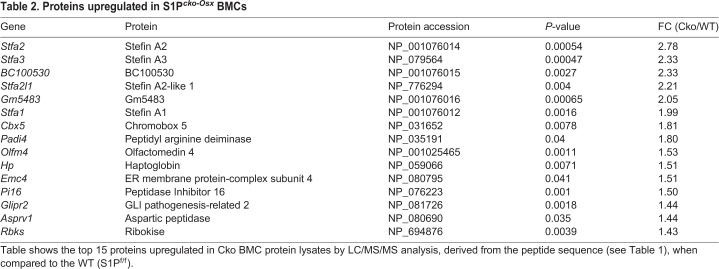
**Proteins upregulated in S1P^*cko-Osx*^ BMCs**

### Single cell analysis of the Cko bone marrow

The *Osx-Cre* transgenic mice expresses a Cre-GFP fusion protein that marks cells in the growth plate of both heterozygote and Cko mice, but only the Cko mice show the presence of GFP^+^ cells in the bone marrow (Patra et al., 2018). The use of *Osx-Cre* mice dictates S1P ablation in Osx-expressing cells, which suggests that GFP^+^ cells have active Osx-Cre activity. This indicates that these cells are relevant and/or instrumental to the phenotype seen in Cko mice. To investigate these cells, we first analyzed the location of these cells in the bone marrow by performing immunofluorescence for endomucin (which identifies endothelial cells in blood vessel lining) to study the relative location of GFP^+^ cells to blood vessels in the bone marrow (Fig. S3). GFP+ cells were often seen in close proximity to endomucin-stained blood vessels (red) in the bone marrow (Fig. S3). Many cell types with mesenchymal stem cell/skeletal stem cell properties have perivascular locations (such as the pericytes), and are important regulatory components of the bone marrow niche, including the hematopoietic stem cell niche (Kunisaki, 2019).

Therefore, to investigate the identity of GFP^+^ cells, we performed Sc-RNA-Seq analysis. Single GFP^+^ cells were FACS-sorted (Fig. S3) into 96-well plates and each cell analyzed by RNA-Seq. Since the absence of S1P in the Osx-lineage induces a decrease in SSCs (defined as triple negative for CD45, Ter-119, CD31, but positive for CD105) in the Cko, we also FACS-sorted SSCs to analyze the relationship between GFP^+^ cells and SSCs by RNA-Seq. To allow for direct comparison of these two cell types, the RNA-Seq dataset from GFP^+^ and SSCs cells [two plates of GFP-sorted Cko BMCs (GFP-Cko1 and GFP-Cko2), two plates for WT SSCs (SSC-WT1 and SSC-WT2) and two plates for Cko SSCs (SSC-Cko1 and SSC-Cko2)] were analyzed together by both Seurat and RaceID clustering tools.

**Fig. 5.**
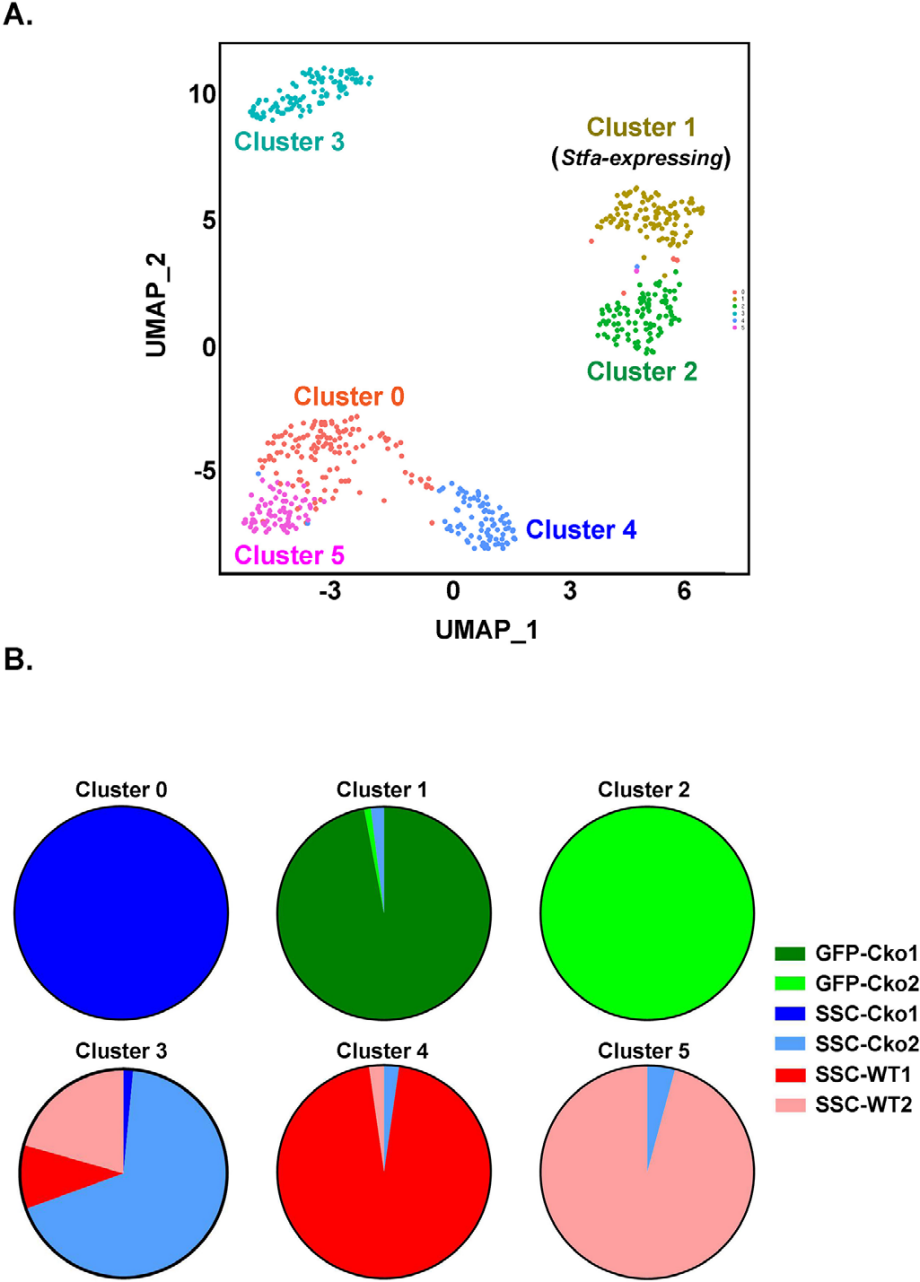
**(A) A UMAP (Uniform Manifold Approximation and Projection) plot from Seurat analysis of Sc-RNA-Seq data from Cko-GFP^+^ cells and WT and Cko SSCs, showing six distinct clusters (0**–**5).** The analysis is based on Sc-RNA-Seq data from six 96-well plates [two GFP-Cko (GFP-Cko1 and GFP-Cko2), two WT SSCs (SSC-WT1 and SSC-WT2)] and two Cko SSCs (SSC-Cko1 and SSC-Cko2). The contribution of each plate to these clusters is shown color-coded in B.

Both Seurat and RaceID clustering tools demonstrated that GFP^+^ cells and SSCs are distinct cell types with very little overlap in their gene expression patterns. Seurat clustering analysis identified six clusters in the UMAP plot (Fig. 5). Clusters 1 and 2, composed primarily of GFP^+^ cells, segregated from the remaining clusters (clusters 0, and 3–5) composed primarily of SSCs. Only cluster 1 cells, composed primarily of cells from GFP-Cko1, with some contribution from GFP-Cko2, demonstrated expression of the *Stfa* gene family (Table 3); cluster 2, composed totally of cells from GFP-Cko2 collection, did not show this trait (not shown). RaceID clustering analysis resulted in five clusters where again the predominant GFP^+^ Cko cells-containing clusters (clusters 1, 4) segregated from clusters containing SSCs (Fig. S4). However, in RaceID clustering analysis, cluster 4 cells, the only cluster whose cells show *Stfa* expression, is represented almost equally with GFP^+^ cells from both GFP-Cko1 and GFP-Cko2 collections (a total of four mice). This indicated strongly that these GFP^+^ cells express stefin proteins. However, both clustering analyses demonstrated that not all GFP-sorted cells express *Stfa*. Overall, the Sc-FACS-Seq and clustering analysis demonstrated that GFP^+^ cells are distinct from SSCs and significantly overexpress the *Stfa* gene family, a characteristic not shown by clusters with predominantly SSCs, whether from WT or Cko. The increased expression of stefins demonstrated by the LC/MS/MS proteomic analysis of the Cko BMCs lysate thus represents expression of stefin proteins by GFP^+^ cells. Both clustering analyses also indicate that SSCs harvested from the Cko segregate both similarly and differentially with WT SSCs. For example, cluster 3, the biggest cluster in the RaceID data set (Fig. S4), is made up of SSCs from both the two WT and two Cko mice indicating their similarities; cluster 5 has Cko-derived SSCs as the primary cell type (75%) along with SSCs from the WT (25%). This similarity and dissimilarity between WT and Cko SSCs is normal considering that CD45, Ter-119, and CD31 triple negative but CD105 positive cells are only enriched for, but are not pure, SSCs.

**Table 3.**
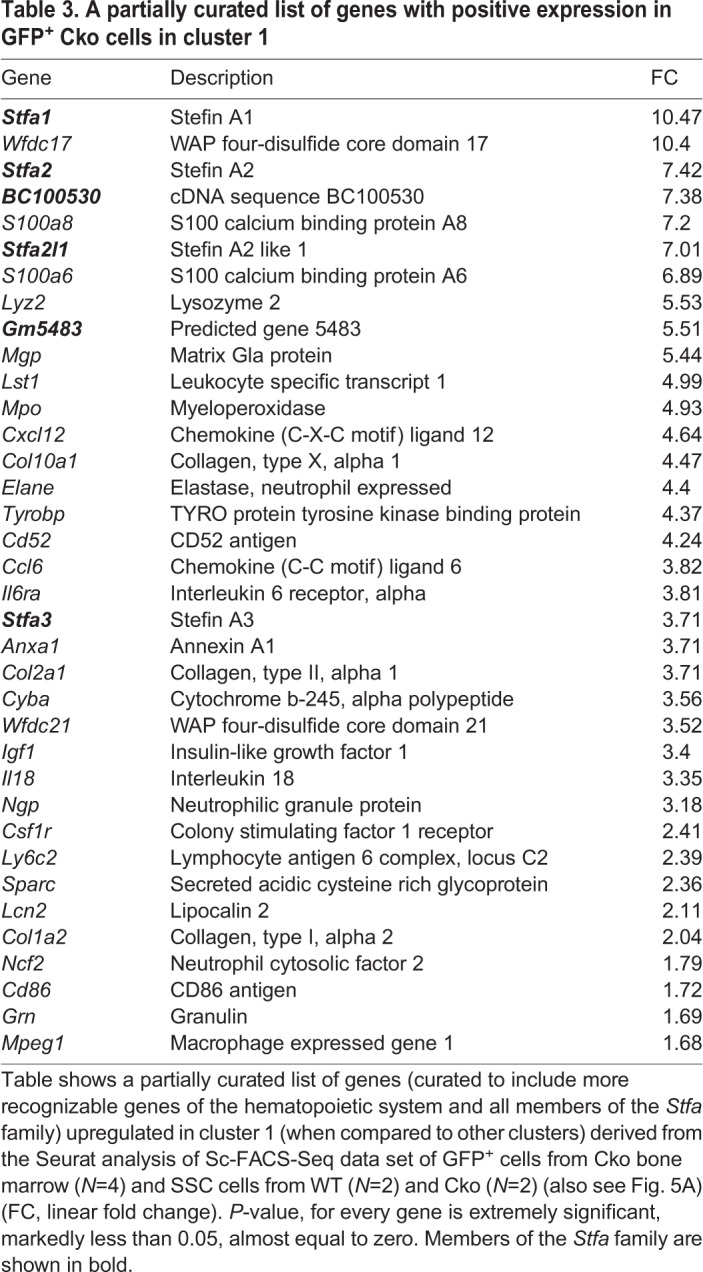
**A partially curated list of genes with positive expression in GFP^+^ Cko cells in cluster 1**

To deduce the identity of GFP^+^ cells, we analyzed the expression profile of cluster 1 from Seurat analysis. Surprisingly, the gene expression list suggested that a majority of GFP^+^ cells were of hematopoietic origin, capable of participating in an inflammatory response as suggested by expression of *Il18*, *Cxcl12* and *Il6ra*, a likely granulocyte/granulocyte-macrophage/leukocyte as suggested by characteristic expression of genes such as *Elane* and *Mpo* (both characteristic of myeloid progenitors) or *S100a8*, *Ngp* and *Lyz2* (all characteristic of neutrophils), or *Csf1r* (characteristic of monocytes and monocyte progenitors), along with *Lst1*, *Ly6c2*, *Tyrobp*, *Grn* and *Mped*, among others (Table 3). In fact, the expression of *Stfa2l1* is considered relatively specific to neutrophils (Ericson et al., 2014). RaceID cluster analysis revealed a similar expression profile (not shown) in cluster 4, which has more prominent single cell contributions from both GFP-sorted plates (GFP-Cko1/2). This suggested that *Osx-Cre* was being expressed in a hematopoietic lineage cell and was not restricted to cells of overt skeletal lineage such as osteoblast progenitors. A study of Table 3 shows the expression of *Mgp*, *Col10a1*, *Col2a1*, *Sparc* and *Col1a2*, characteristic of an expression pattern that belongs to a skeletal lineage, though this does not appear to be the primary phenotype. It is highly probable that GFP*^+^* cells sorted are of a mixed lineage, i.e. skeletal and hematopoietic with the majority of GFP-expressing cells in the bone marrow being of hematopoietic origin. In summary, the identification of Stfa proteins (upregulated as per proteomics, see above) in GFP-expressing hematopoietic cells in the Cko bone marrow suggest that they are important regulators of the mutant phenotype.

### Global analysis of BMCs via the 10x Genomics platform

Both the proteomic and Sc-FACS-Seq analysis revealed the significant upregulation of the *Stfa* gene family, but surprisingly the single cell analysis of GFP-sorted cells demonstrated that this was likely happening in hematopoietic cells because of the overwhelming association of leukocyte/macrophage-associated genes along with stefin expression in the cluster. Therefore, to get a global understanding of how the bone marrow environment is altered in the Cko, we performed single cells analysis of a large number of WT and Cko non-RBC BMCs using the 10x Genomics platform (www.10xgenomics.com). Data for the WT was extracted from the sequencing of 6287 single cells averaging 27,316 reads/cell with 1633 median genes/cell analyzed, whereas 3670 single cells were sequenced for the Cko with 46,326 reads/cell with 1698 median genes/cell analyzed. Seurat analysis produced ten unique tight clusters for both WT (Fig. S5) and the Cko (Fig. S6) indicating analysis of adequate and sufficient cell numbers.

To identify cells in each cluster and determine their prevalence and equivalence of Cko with WT, we analyzed the number of cells in each cluster (Table 4) and compared their positive gene expression profile. These data indicated that the Cko bone marrow deviated from the WT in some key aspects. Of the ten clusters, seven Cko clusters showed excellent equivalence with WT clusters, notably cells in Cko cluster 1 (with cluster 3 in WT), cluster 3 (with cluster 4 in WT), cluster 4 (with cluster 0 in WT), cluster 6 (with cluster 6 in WT), cluster 7 (with cluster 7 in WT), and cluster 8 (with cluster 5 in WT) (Tables 4,5,6; Table S3) where their gene expression profile matched almost identically with the WT. However, there were notable differences. Clusters 2, 8 and 9 in the WT do not have equivalent clusters in the Cko; likewise, clusters 2, 5 and 9 in the Cko do not have a corresponding cluster in the WT. The most striking observation is the duplication of the *Stfa1*- and *BC100530-*expressing (both members of the *Stfa* family) *Ly6G* population in the Cko, namely clusters 0 and 2, which identifies these populations as neutrophils (Table 5). In the WT, only one cluster, cluster 1 (17.61% of the population in clusters, Table 4) is seen that has a gene expression profile characteristic of neutrophils, notably *Ly6G*-expressing (a marker for neutrophils), and the high expression of *Lcn2* [also known as neutrophil gelatinase-associated lipocalin (NGAL)], *Ltf*, *Ngp* and *S100a8* among others. This cluster shows the expression of *Stfa1* and *BC100530* genes only, indicating that the expression of these genes in neutrophils is normal (Table 5). Cluster 0 in the Cko (15.67% of the Cko BMCs, Table 4) has a similar expression profile, but characterized by the additional expression of *Stfa2*, *Stfa2l1* and *Stfa3*, not seen in WT cluster 1 (Table 5). The Cko, however, has an additional cluster, cluster 2 (15.29%), which has a similar expression profile to both WT cluster 1 and Cko cluster 0, is *Ly6G*-expressing, but is characterized by the expression of more members of the *Stfa* gene family, namely *Gm5483* and *Stfa2l1*, in addition to expression of *Staf1*, *BC100530*, *Stfa2* and *Stfa3*. Thus, in the Cko, clusters 0 and 2 appear to be essentially *Ly6G*-expressing neutrophil clusters and account for 30.96% of the population, close to twice the population seen in the WT. Importantly, these two clusters are the only clusters that show expression of the *Stfa* gene family, and in this respect are similar to the expression profile of GFP-sorted cluster 1 cells (see Table 3, Fig. 5), the only cluster with expression of the *Stfa* gene family. These observations indicate that the GFP-sorted Cko cells from cluster 1 (Table 3, Fig. 5) and cells from WT cluster 1 and Cko clusters 0 and 2 (Table 5) from the 10x Genomics Sc-RNA-Seq analysis are identical; these cells are likely leukocytes, namely neutrophils. Notably, neutrophils in the Cko also appear to be biologically different from those in the WT due to the different expression profile of the *Stfa* family.

**Table 4.**
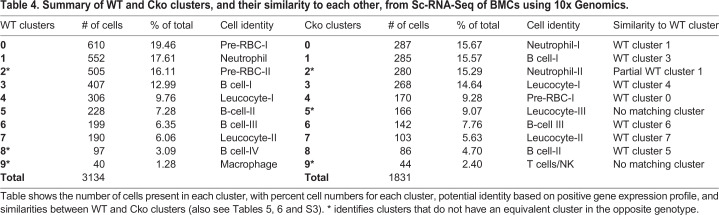
**Summary of WT and Cko clusters, and their similarity to each other, from Sc-RNA-Seq of BMCs using 10x Genomics.**

**Table 5.**
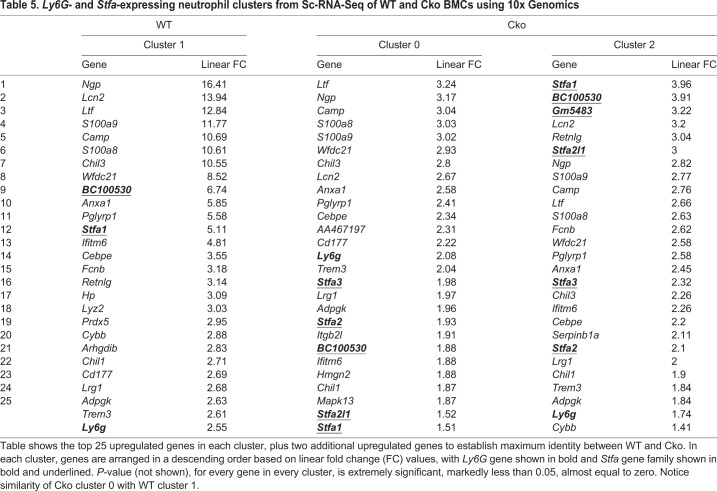
***Ly6G*- and *Stfa*-expressing neutrophil clusters from Sc-RNA-Seq of WT and Cko BMCs using 10x Genomics**

**Table 6.**
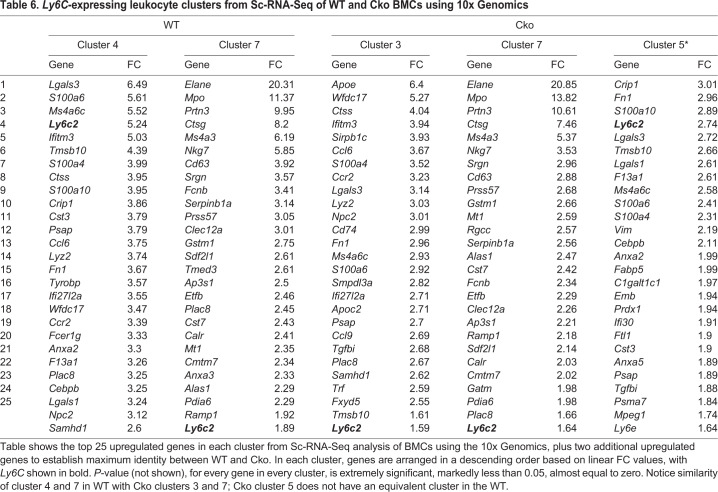
***Ly6C*-expressing leukocyte clusters from Sc-RNA-Seq of WT and Cko BMCs using 10x Genomics**

The Cko bone marrow showed strong evidence of a pro-inflammatory disposition. Cluster 9 in the Cko has an expression profile characteristic of T cells/NK cells as marked by the expression of *Ccl5* (Rantis), *Gzma* (Granzyme), *Cd3d*, *Cd3g*, *Cd3e* and *Thy1* to mention a few, a cluster that is absent in the WT (Table S3). Furthermore, there are at least three *Ly6C*-expressing clusters in the Cko (clusters 3, 5 and 7), but only two *Ly6C*-expressing clusters (clusters 4 and 7, identical to clusters 3 and 7 in the Cko, respectively) in the WT (Table 6). Thus, cluster 5 in the Cko is an additional *Ly6C*-expressing cluster that maybe an early progenitor stage or a different maturation stage than the WT. Notably, cluster 3 in the Cko makes up 14.64% of the total cluster population, as compared to its corresponding cluster in the WT, cluster 4, which is only 9.76%, though clusters 7 in the WT and Cko are very similar in their prevalence. Though neutrophils and monocytes both express *Ly6C* (Lee et al., 2013), the lack of any similarity in the expression profile of the *Ly6C* clusters with the *Ly6G*-expressing clusters suggests that there is also an increase in monocytes or other *Ly6C*-expressing leukocytes such as T cells, NK cells and dendritic cells in the Cko. The WT bone marrow also showed the presence of other non-leukocyte clusters that were lacking in the Cko (Table S3). Notably here, Cko has one less cluster that could be characterized as a B cell cluster based on its expression profile, suggesting less B cell numbers in the Cko bone marrow.

Thus, the combined proteomic and Sc-RNA-Seq analysis revealed that *Osx-Cre* transgene is likely active in hematopoietic cells (neutrophils) suggesting S1P ablation in the hematopoietic lineage; furthermore, S1P ablation in the Osx-lineage causes a doubling of bone marrow neutrophils and other leukocytes. More importantly, these analyses also suggest cell-intrinsic changes as demonstrated by neutrophils which express more and additional members of the stefin proteins; the dramatic increase in stefins allude to a causal role for stefins in the bone phenotype (osteopenia) in the Cko mice.

### Hematopoietic alterations in the Cko bone marrow

**Fig. 6.**
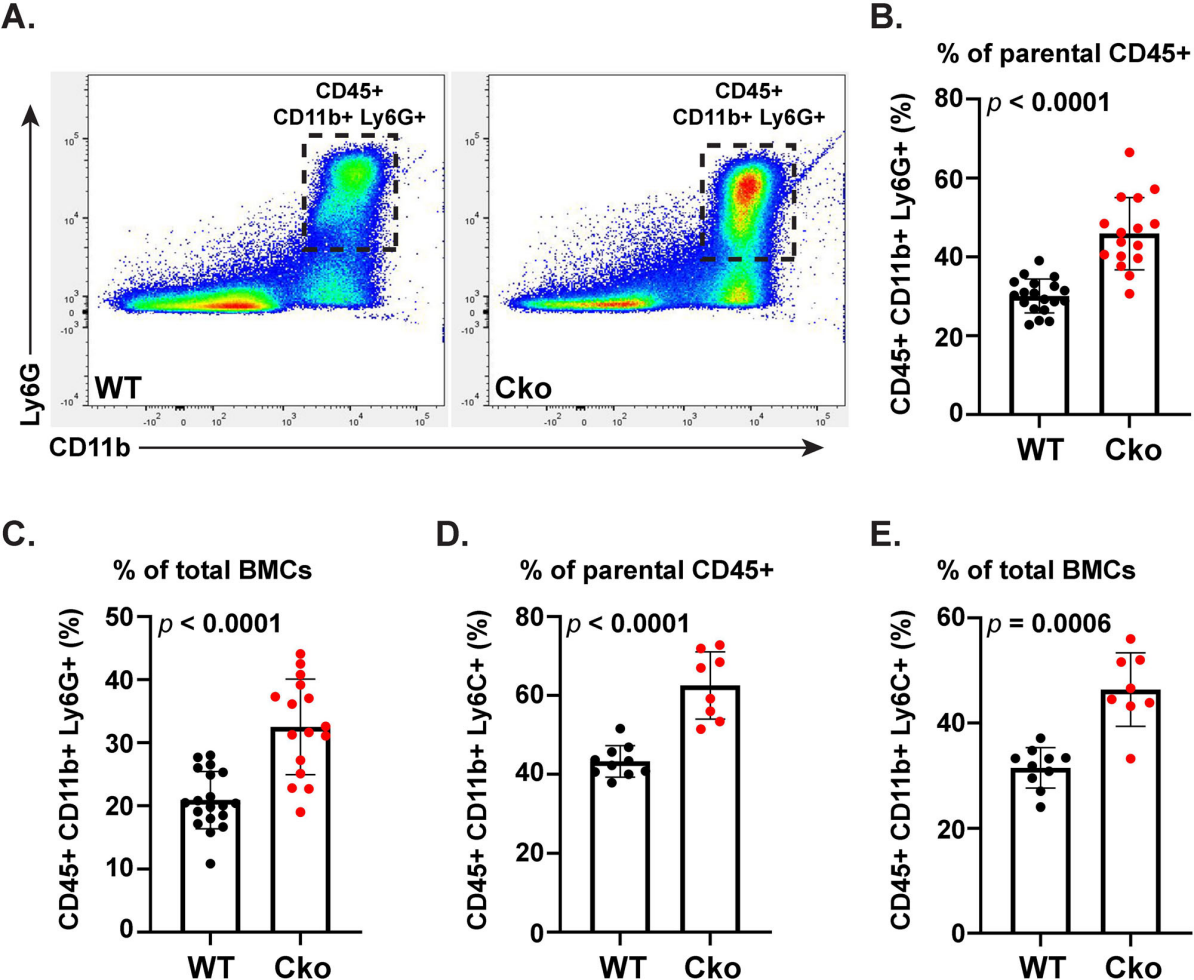
**FACS analysis of neutrophils in WT and Cko bone marrow.** (A) A flow cytometry dot plot of CD11b^+^ Ly6G^+^ double-positive cells in WT and Cko BMCs gated from CD45^+^ population is shown (also see Fig. S7). A typical representative plot from several experiments is shown. (B,C) Quantitation of CD45^+^ CD11b^+^ Ly6G^+^ triple-positive cells in WT and Cko bone marrow (*N*≥16). Data are shown as percent of parental CD45^+^ (B) or total cells (C). (D,E) Quantitation of CD45^+^ CD11b^+^ Ly6C^+^ triple-positive cells in WT and Cko bone marrow (*N*≥8). Data are shown as percent of parental CD45^+^ (D) or total cells (E). The *P*-values for the comparison between WT and Cko were calculated using non-parametric *t*-test (two-tailed) with Mann–Whitney correction from the analyses of WT and Cko mice from several different litters.

To investigate the increase in the neutrophils and their association with stefin proteins, we performed FACS analysis of BMCs from WT and Cko using fluorescent-conjugated antibodies against established neutrophil markers in association with an antibody for stefin. Using fluorescent-conjugated antibodies we assayed CD45^+^ CD11b^+^ Ly6G^+^ triple-positive population, which is a hallmark of neutrophils (Swamydas et al., 2015), in WT and Cko bone marrow (Fig. 6; Fig. S7). The Cko bone marrow showed a significant and reproducible marked increase in CD45^+^ CD11b^+^ Ly6G^+^ triple-positive population over the WT, both in terms of the number of neutrophils present in the CD45^+^ myeloid population or as a percent of total BMCs analyzed, over many different litters (Fig. 6A–C). In agreement with the Sc-RNA-Seq analysis above, the Cko bone marrow has approximately twice the number of CD45^+^ CD11b^+^ Ly6G^+^ triple-positive neutrophils than the WT. Next, we analyzed how many of these triple positive cells also expressed stefin proteins. Our analysis show that 100% of CD45^+^ CD11b^+^ Ly6G^+^ neutrophils also stained positive for stefins in both WT (Fig. S7G,H) and Cko (not shown). Thus as expected based on cluster analysis (Table 5) that showed *Stfa* expression in *Ly6G*-expresing neutrophils in both WT (cluster 1) and Cko (clusters 0 and 2) BMCs, FACS analysis also demonstrated the co-localization of stefin expression with CD45^+^ CD11b^+^ Ly6G^+^ neutrophils. We also analyzed the increase in the Ly6C^+^ population by FACS. Specifically, we analyzed for the CD45^+^ CD11b^+^ Ly6C^+^ triple-positive leukocytes and found that like the neutrophils, the Cko bone marrow showed a significant upregulation of Ly6C^+^ positive cells both in the CD45^+^ myeloid population (Fig. 6D) and as part of total BMCs analyzed (Fig. 6E).

**Fig. 7.**
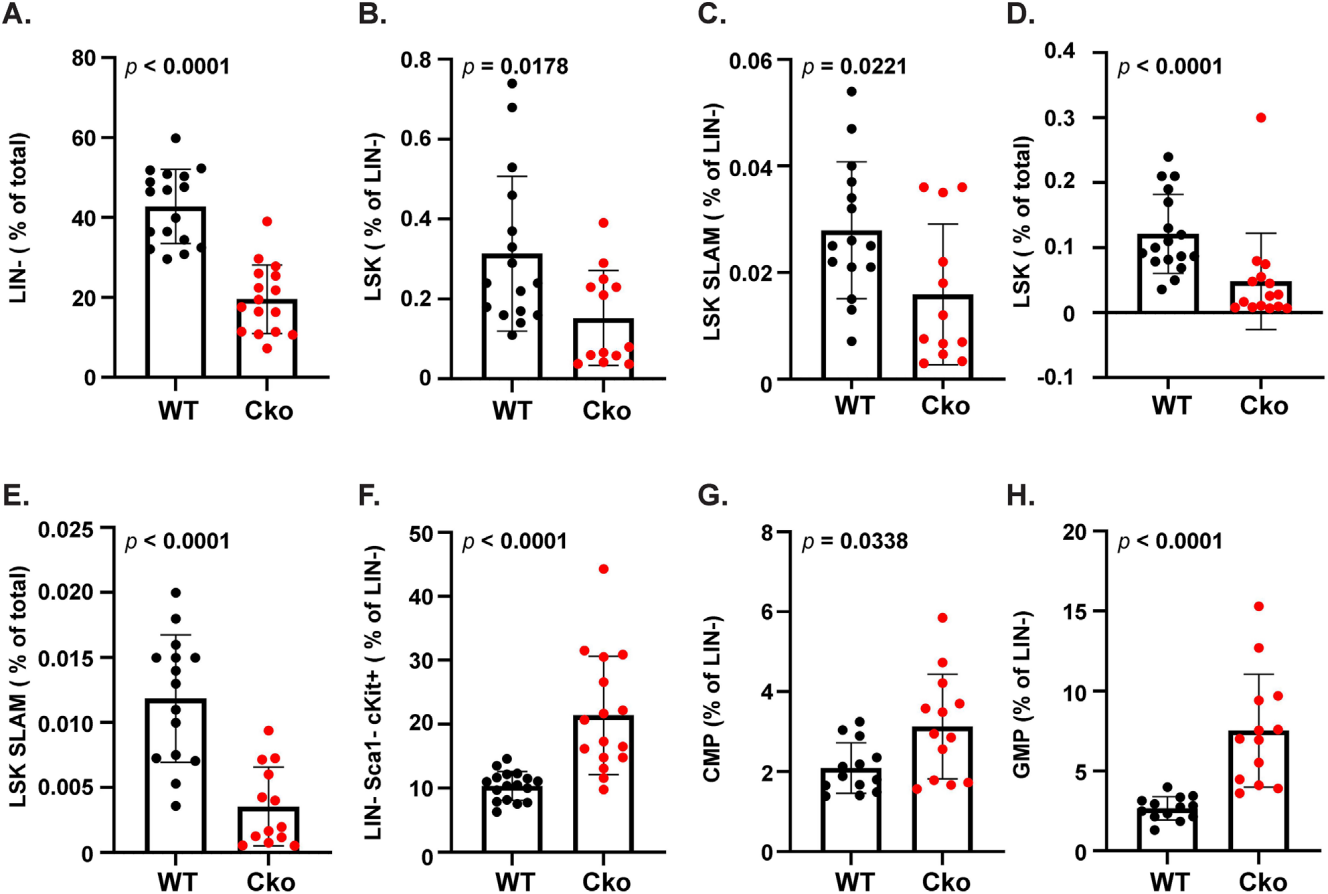
**FACS analysis of hematopoietic stem cells in WT and Cko mice.** (A) Graph showing the decrease in LIN^−^ cells in the bone marrow of Cko mice when compared to WT. Data are shown as percent of total BMCs analyzed. Graphs showing LSK (B, % of LIN^−^), LSK SLAM (C, % of LIN^−^), LSK (D, % of total), LSK SLAM (E, % of total), LIN^−^ Sca1^−^ c-Kit^+^ (F, % of LIN^−^), and the LIN^−^ Sca1^−^ c-Kit^+^ derived CD34^+^ CD16/32^−^ (CMP) (G, % of LIN^−^) and CD34^+^ CD16/32^+^ (GMP) cells (H, % of LIN^−^) in the bone marrow of WT and Cko mice. The *P*-values for the comparison between WT and Cko were calculated using non-parametric *t*-test (two-tailed) with Mann–Whitney correction from the analyses of WT (*N*≥12) and Cko (*N*≥12) mice from several different litters.

To investigate if this increase in leukocytes could be traced to alterations in hematopoietic stem cells in the Cko, we conducted FACS analysis of different populations of hematopoietic progenitors (Fig. S8; Fig. 7). Multipotent hematopoietic stem cells (HSCs) reside in the bone marrow that either self-renew or differentiate into lineage (LIN)-restricted progenitors, which give rise to differentiated hematopoietic cells such as leukocytes/neutrophils. Multi-lineage HSCs are often defined by negative selection for markers of differentiated hematopoietic lineages (LIN^−^) and positive selection for cell surface markers Sca1 and c-Kit (Fig. S8). As compared to the WT, the Cko bone marrow showed a significant decrease in total non-differentiated LIN^−^ stem cells (Fig. 7A), which suggested a decrease in multipotent HSCs. In keeping with this observation, the Cko demonstrated a significant decrease in the long-term, multipotent LSK stem cell population that is enriched for HSCs when compared to the WT, not only in relation to the total LIN^−^ cells (Fig. 7B) but also in comparison to total BMCs analyzed (Fig. 7D). The LSK population is only considered enriched for HSCs where only 10% of this population is considered legitimate multipotent HSCs. Therefore we analyzed the LSK population further by combining markers for the SLAM family of cell surface glycoproteins CD150 and CD48, where approximately 50% of LSK-SLAM (LSK CD150^+^ CD48^−^) cells are expected to have true HSC activity (Challen et al., 2009). Like the LSK population, the Cko bone marrow also demonstrated a significant downregulation of the LSK-SLAM stem cells (Fig. 7C,E).

Within the LIN^−^ population, the more differentiated, lineage-restricted progenitors such as the megakaryocyte-erythrocyte progenitor (MEP), common myeloid progenitor (CMP), and the GMP are characterized as c-Kit^+^ but Sca1^−^. Remarkably, the LIN^−^ Sca1^−^ c-Kit^+^ population is significantly upregulated in the Cko (Fig. 7F), which suggested deviations from the WT in the MEP, CMP and GMP progenitors. The CMP progenitors and its derivative GMP are precursors to leukocytes (neutrophils/monocytes) (Seita and Weissman, 2010). When we further analyzed the LIN^−^ Sca1^−^ cKit^+^ population with CD34 and CD16/32 markers, CMP (LIN^−^ Sca1^−^ cKit^+^ CD34^+^ CD16/32^−^) (Fig. 7G) and especially GMP (LIN^−^ Sca1^−^ cKit^+^ CD34^+^ CD16/32^+^) (Fig. 7H) progenitors were significantly upregulated in the Cko bone marrow. GMPs were on average more than twice upregulated in the Cko, which correlates well with the doubling of neutrophils and Ly6C^+^ population in the Cko bone marrow (Fig. 6). Though the MEP population remained unaffected, the LIN^−^ Sca1^−^ cKit^+^ CD34^−^ CD16/32^+^ population was remarkably upregulated in the Cko bone marrow (Fig. S9), though the significance of this population remains unknown. Thus, S1P has a role in the specification of HSCs and/or hematopoietic progenitors to the correct lineage; the absence of S1P results in a shift to the myeloid lineage with more leukocyte production. S1P thus maintains both SSCs (Patra et al., 2018) and HSCs and our data endorses the importance of S1P in maintaining the balance and reciprocal relationship of these two different kinds of stem cells in the bone marrow.

### Stefin proteins and the skeletal system

**Fig. 8.**
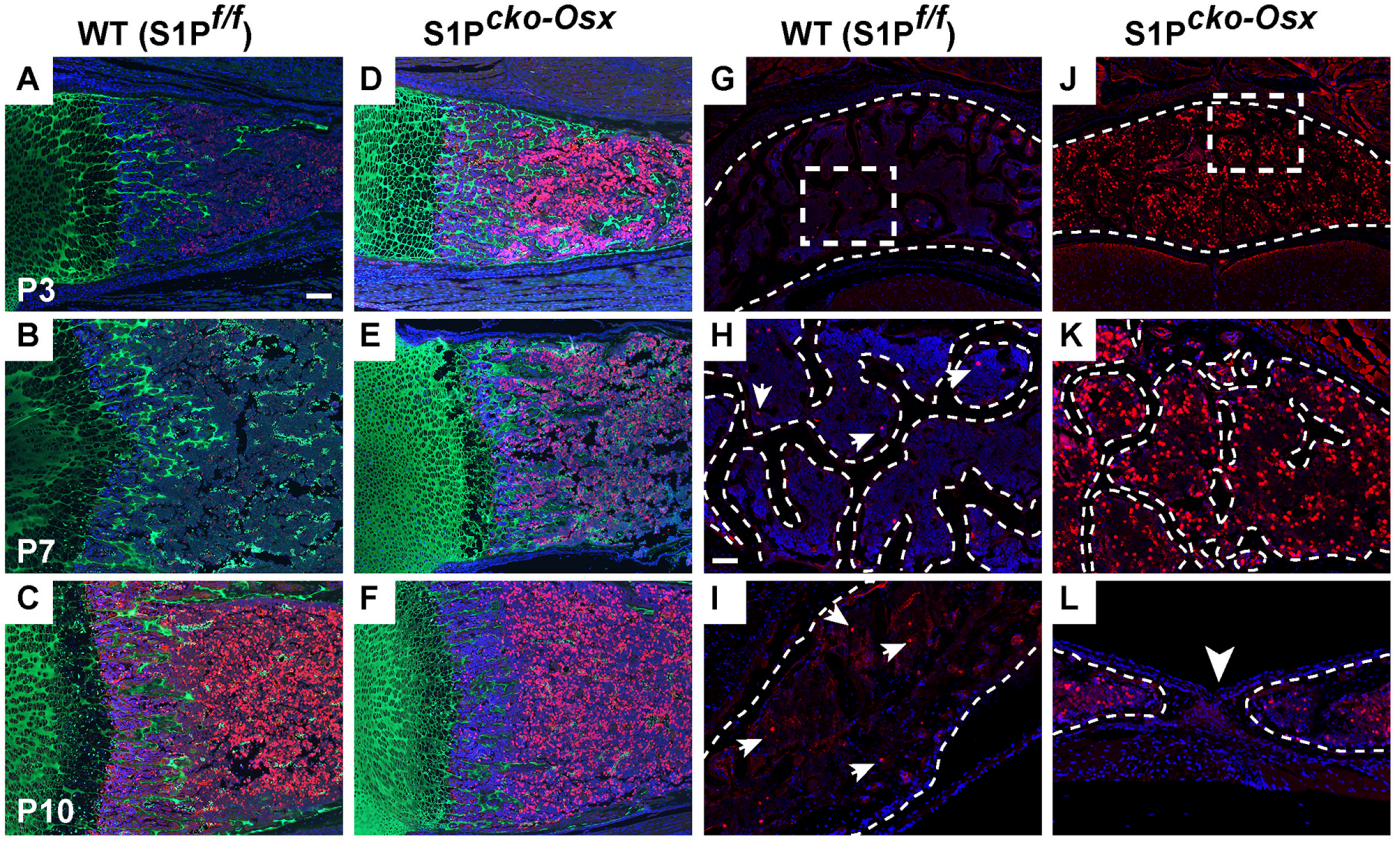
**IF for stefin expression in WT and Cko mice.** Figure shows IF for stefin (red) in P3 (A,D), P7 (B,E) and P10 (C,F) femora (A–F) and in P21 C2 (Axis) vertebrae of the spine (G–L) in WT and Cko mice. IF for stefin in the femora (A–F) is shown as doubled-labeled with type II collagen (green) to demarcate cartilage. Panels G–K show staining for stefin in the posterior arch of C2 where H and K are higher magnification images of the outlined regions in G and J, respectively; panels I and L show stefin expression in the neural arch with the arrowhead in L pointing to the SBO in the Cko. Outlined regions in H and K demarcate bone trabeculae from the bone marrow. The outlined regions in I and L demarcate the neural arch from the surrounding tissue. Arrows in panels H and I point to the limited stefin expression in the WT. Blue: DAPI-stained nuclei. Scale bars: (A–J): 100 µm; (H–L): 50 µm.

To understand how stefin expression correlated with bone, cartilage and bone marrow compartments *in vivo*, we performed immunofluorescence (IF) analysis for stefins in WT and Cko femora and vertebrae, specifically C2 (Axis) (Fig. 8). We chose C2 because SBO is most commonly seen in these vertebrae in our studies, and to understand stefin expression in relation to SBO occurrence. In the femora, we performed double-labeled IF analysis for type II collagen (Col II) (green) and stefin (red) to demarcate the cartilage and study stefin expression in relation to cartilage (Fig. 8A–F). Our studies above done with P21 mice already demonstrated the increased expression of the Stfa proteins in the Cko when compared to the WT. So in this IF analysis we studied stefin expression in femora of younger mice, notably P3, P7 and P10. IF analysis demonstrated that stefin expression is seen in both WT and Cko, but restricted to the bone marrow. Stefin expression was not seen in the cartilaginous areas unless it was also traversed by bone marrow as in the cartilaginous regions next to trabecular bone. Nor was stefin expression detected in solid bone. Interestingly, stefin is visibly upregulated in the Cko bone marrow as early as P3 and continued to P10. Stefin expression in the P21 WT C2 bone marrow is seen occasionally (arrows in Fig. 8G,H,I). However, its expression in the Cko C2 vertebrae is remarkably high when compared to the WT (Fig. 8J,K). Here too, its expression is restricted to the marrow compartments, and notably absent from bone trabeculae or any other tissue, including the connective tissue between the unfused bones of the neural arch in the Cko (arrow, Fig. 8L).

**Fig. 9.**
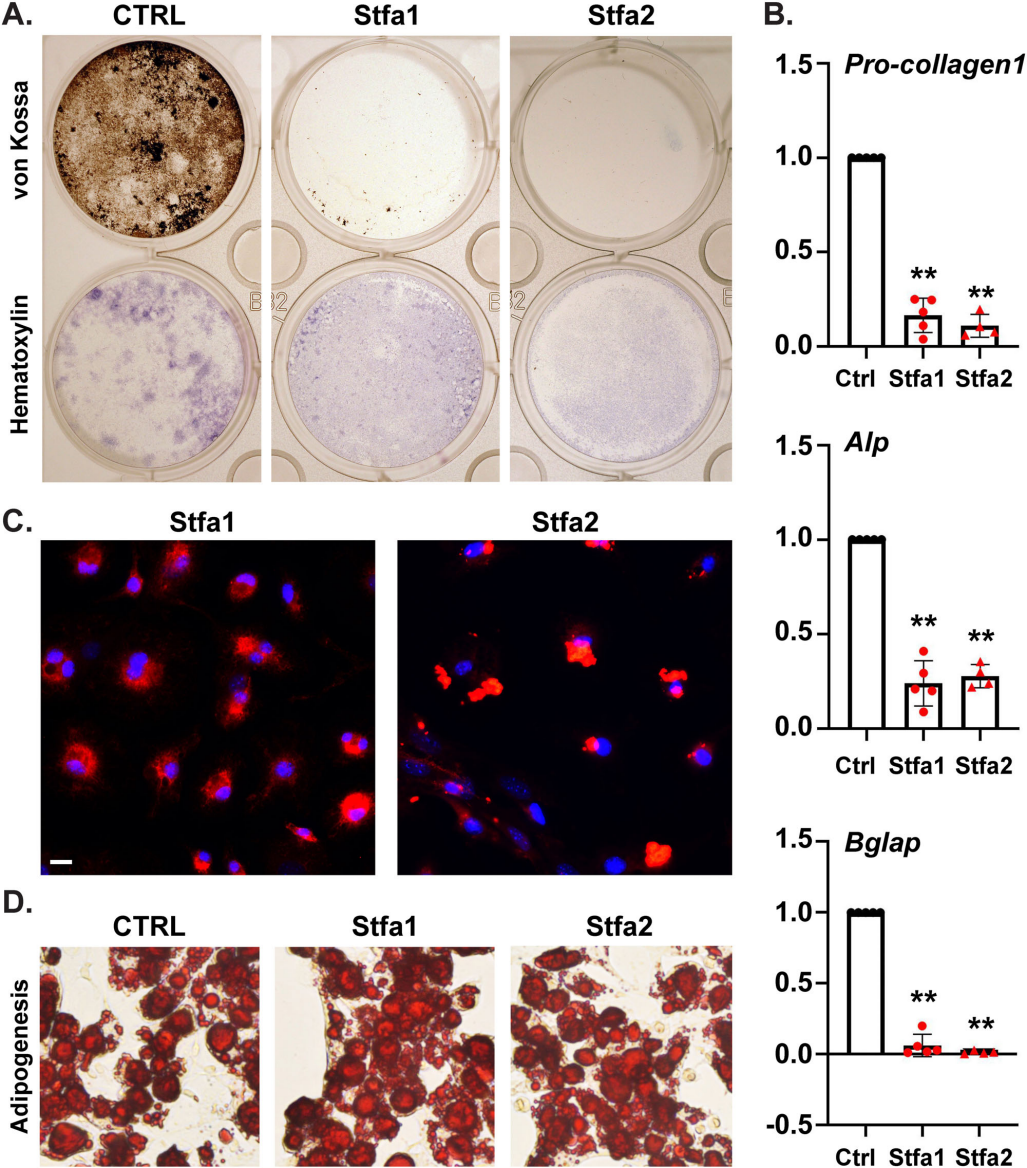
**Stefin protein selectively inhibits osteogenesis.** (A) *In vitro* osteogenic differentiation assays with BMSCs from P21 WT (S1P*^f/f^*) mice with or without (CTRL) His6-Stfa1 or His6-Stfa2 proteins at 250 ng/ml. Typical results are shown from *N*=4 at day 14. Assays were done in duplicate with upper panels stained for von Kossa and lower panels with Hematoxylin. (B) qPCR analysis at day 7 for *Pro-collagen1* (procollagen type I), *Alp* (alkaline phosphatase) and *Bglap* (osteocalcin) with RNA harvested from WT BMSCs treated with 250 ng/ml of His6-Stfa1 or His6-Stfa2 (mean±s.d.; *N*=4-5; **P=0.0079). (C) Immunocytochemistry for His6-tag at day 3 on BMSCs treated with 250 ng/ml of His6-Stfa1 or His6-Stfa2 (Red=His6 tag; Blue=DAPI-stained nuclei). Scale bar: 50 µm. (D) *In vitro* adipogenic differentiation assays with BMSCs from P21 WT (S1P*^f/f^*) mice with or without (CTRL) His6-Stfa1 or His6-Stfa2 proteins at 500 ng/ml. Typical results are shown from *N*=4 at day 7.

Next, we analyzed if stefins could regulate osteogenic differentiation, *in vitro*. For this, we performed *in vitro* osteogenic differentiation assays using bone marrow stromal cells (BMSCs) harvested from WT (S1P*^f/f^*) mice in the presence and absence of 6-histidine tagged stefin 1 and stefin 2 (His6-Stfa1/Stfa2) proteins. Remarkably, the addition of His6-Stfa1 or His-Stfa2, as low as 250 ng/ml, dramatically inhibited osteogenic differentiation of BMSCs (Fig. 9A). Hematoxylin staining of cells in the assay show the presence of live cells, which suggests no reduction in cell numbers due to the addition of stefin in the assay, but only an inability to undergo osteoblast differentiation. This was confirmed further by qPCR analysis at day 7 for *Pro-collagen 1*, *Alp* and *Bglap* genes that were significantly downregulated on addition of His6-Stfa1 or His6-Stfa2 (Fig. 9B). To confirm that this was due to the transport of stefins inside BMSCs, we performed IF analysis on the cells for the recombinant proteins using an antibody for the 6-His-tag. After only 3 days of incubation with stefins, BMSCs showed the ability to uptake both His6-Stfa1 and His6-Stfa2 (Fig. 9C) from the media. While His6-Stfa1 showed a diffuse intracellular distribution, His6-Stfa2 is restricted to discrete locations inside the cell. To investigate if the inhibitory effect of stefins is specific to osteogenesis, we analyzed the effect of stefins on adipogenesis *in vitro*. Interestingly, neither His-Stfa1 nor His6-Stfa2 showed any ability to inhibit adipogenesis even at 500 ng/ml (twice the concentration that inhibited osteogenesis) (Fig. 9D), indicating that stefins selectively regulate osteogenesis, but not adipogenesis.

The Cko neutrophils showed not only an increase in stefin expression, but also the expression of more stefin family members (Table 5). To analyze if this could be due to the direct ablation of S1P exon 2 (the floxed exon in the S1P gene, *Mbtps1*) in Cko neutrophils, we FACS-sorted CD45^+^ Ly6G^+^ CD11b^+^ triple-positive neutrophils from WT and Cko mice and analyzed for the presence of *Mbtps1* exon2 by sequencing. Neutrophils from six Cko mice were analyzed and all six mice showed ablation of *Mbtps1* exon 2 (Fig. S10)*.* This data suggested that S1P ablation is directly responsible for the increase in bone marrow neutrophils and for their intrinsic changes in stefin expression in the Cko.

## DISCUSSION

In our previous study with this mouse model we had established that S1P ablation in the Osx-lineage (S1P*^cko-Osx^* or Cko mice) results in varying degrees of scoliosis along with a drastically compromised bone development program that results in osteopenia (Patra et al., 2018). The presence of scoliosis and other skeletal abnormalities in a human patient with drastically reduced S1P expression further underscores the importance of S1P for normal mammalian skeletal development (Kondo et al., 2018). In this study, we established further the importance of S1P, not only to skeletal homeostasis, but also in maintaining the regenerative capacity of the bone marrow niche where both skeletal and hematopoietic progenitors reside, and are produced.

Our in-depth characterization of scoliotic spines show that these mice also have SBO. Hyperplastic fracture calluses at the costovertebral articulations were also seen in association with scoliosis and these fractures were present only on one side of the spine. Spontaneous fractures are common in mouse models that show defects in osteoblast activity such as *Opt-/-* mice in which fracture callus formation is seen on both sides (Sohaskey et al., 2010), and mutant *Sfx* mice with vitamin C deficiency (Mohan et al., 2005), all hallmarks of osteopenia. The S1P*^cko-Osx^* (Cko) mice also have impaired osteogenesis, coupled to spontaneous fractures, with downregulation of bone development. These observations suggest that hyperplastic fractures in the Cko are a consequence of impaired bone formation; however, the asymmetrical nature of callus occurrence at the costovertebral articulations may be imposed by the bending of rib cage due to twisting of the spine. As the fracture is always seen on the side of ribs that is maximally twisted, the twisting of the spine likely imposes additional stress that synergizes with impaired bone formation to fracture the bone at this junction.

Scoliosis is considered to be multifactorial and often of unknown etiology (idiopathic). However, our mouse model suggests that low BMD could be an etiological factor for scoliosis and this association is seen in humans (Li et al., 2008). While the etiology of scoliosis remains complex, the advent of SBD in our mouse model can be traced to the lack of S1P functions. During development of a vertebral body, two secondary ossification centers (SOC) are formed at the tips of the transverse processes of a vertebral body and a third SOC at the tip of the spinous process in the posterior arches. The ossification at this latter SOC drives the fusion of the two posterior arches to form the spinous process and a canal that encompasses the spinal cord. Our previous studies have shown that lack of S1P in mice delays or prevents the formation of the SOC in the appendicular elements (Patra et al., 2011, 2018). Thus, S1P ablation in the Osx-lineage would prevent or delay SOC formation at this critical juncture to prevent ossification and fusion of the two posterior arches, resulting in SBO. Our study therefore suggests that the advent of scoliosis can compound spine abnormalities and induce additional aberrations such as SBO in the context of an impaired bone formation program.

Our investigations to uncover the underlying molecular aberrations for these phenomena in the Cko mice demonstrated an overwhelming association of this mutant phenotype with the doubling of bone marrow neutrophils and with a family of proteins called stefins. Stefins (also referred to as type I cystatins), discovered in both humans and mice, are part of the cystatin protein family. The mouse genome has at least four known stefin A variant genes, namely *Stfa1*, *Stfa2*, *Stfa2l* (*Stfa2a-like*) and *Stfa3*, and are orthologs of the human stefin A (Mihelič et al., 2006; Mezzapesa et al., 2019); all four *Stfa* variants are over-expressed in the Cko. Stefins are small monomeric proteins of ∼100 amino acids, primarily intracellular, cytosolic proteins and are known inhibitors of cathepsins (Turk et al., 2012). The human stefin A has been studied in association with malignant tumor progression and metastasis. In hepatocellular carcinoma, significantly higher stefin A expression at both mRNA and protein level was seen in malignant tissue as compared to normal hepatic tissue with a significant positive association to tumor size and node metastasis (Lin et al., 2016). In a study on breast cancer, stefin A evolved as a suppressor of early tumor invasion with normal breast tissue showing higher expression for stefin A than tumor tissue (Duivenvoorden et al., 2017). Stefin A was also upregulated in the murine megakaryocyte and platelets in humans and mice with type 2 diabetes (Mezzapesa et al., 2019).

Neutrophils normally express stefin (Davies and Barrett, 1984) and this is also seen in our study as CD45^+^ CD11b^+^ Ly6G^+^ neutrophils from WT mice show stefin expression, also confirmed by IF studies. However, stefins are overexpressed in the Cko, with the doubling of the neutrophil population contributing to more stefin expression. More importantly, expression of additional members of the stefin family is seen in the neutrophils that indicates important cell-intrinsic changes. Thus, these neutrophils could be biologically different than those seen in the WT that could drive the Cko phenotype. Characterization of the hematopoietic lineages in mice with S*tfa2l1* deletion showed increased expression of *Stfa1*, *Stfa2*, *Stfa3* genes in hematopoietic tissues without any increase in the neutrophil population, or changes in long-term repopulating LSK hematopoietic stem cell lineages (Bilodeau et al., 2009). This indicates that S1P deletion in the Osx-lineage is directly responsible for the decrease in LSK and LSK SLAM stem cells; the loss of S1P is also responsible for the skewed increase in neutrophils that can be traced to the increase in CMP and GMP progenitors. The impact of loss of S1P is profound in that GMP progenitors increase despite the drastic overall decrease in LIN^−^ cells. The decrease in LIN^−^ cells could be attributed to the decrease in HSCs; but it could also be attributed to a rapid shuttling of LIN^−^ cells to the more differentiated GMPs and eventually to neutrophils in the absence of S1P. Thus, S1P functions to maintain homeostasis of the hematopoietic regenerative environment in the bone marrow niche.

Published data suggest that stefin overexpression is usually associated with a diseased state of a tissue. Similarly, our study demonstrates that stefin overexpression correlates directly with abnormal alterations of the bone marrow, a bias towards myeloid differentiation and the suggested decrease in lymphopoiesis (B cells), and also correlates negatively with bone development. Given the increase in inflammatory components in the Cko bone marrow, inflammatory crosstalk in the bone marrow could be damaging to stem cell production and/or maintenance. This could be responsible for the decrease in SSCs and osteogenic differentiation seen in these mice (Patra et al., 2018). Osteoblasts are known to support B cell development and differentiation from HSCs (Wu et al., 2008; Zhu et al., 2007). The likely decrease in B cells in the Cko bone marrow thus could be attributed to the downregulation in osteogenic differentiation and bone growth in these mice, endorsing the reciprocal relationship between osteoblastogenesis and hematopoiesis. Neutrophils are known to play a role in inflammation-induced bone loss (Hajishengallis et al., 2016) and secrete chemokines and cytokines such as CCL2 and CCL20, which act as immunomodulatory factors. The doubling of neutrophils in the Cko could create a positive inflammatory loop in the bone marrow exacerbating conditions for stem cell production/survival. Thus, this environment is dysfunctional to normal hematopoiesis and could induce myeloproliferative disorders and support disease induction and progression.

The Osx protein is required for the production of osteoblasts (Nakashima et al., 2002) and this doctrine suggests that the Osx promoter is active only in skeletal progenitors, which excludes the hematopoietic lineage. In contrast, our findings suggest that this promoter is also active in the hematopoietic lineage. The GFP expression observed in the neutrophils/granulocytes/leukocytes (as suggested by Table 3) indicate Cre-GFP expression in these cell types (or in their progenitors), suggesting S1P ablation in these myeloid cells. In our previous study we had well established the efficiency of the *Osx-Cre* transgene in ablating S1P, and its overlap with GFP expression (Patra et al., 2018). This therefore suggests that *Osx-Cre* is active in myeloid progenitors possibly due to normal osterix expression in this lineage. Indeed, a study by Drs Biancamaria Ricci and Roberta Faccio has demonstrated that the osterix protein is expressed in hematopoietic stems cells and also in the more differentiated progenitors such as the GMPs, but not in mature neutrophils (personal communication). This suggests normal activation of the *Osx-Cre* promoter in myeloid precursors, and not due to an artifact of artificial promoter construction in creation of the *Osx-Cre* transgenic mice. Thus, the myeloid anomalies seen in this study are possibly a direct effect of S1P ablation in the hematopoietic lineage. Indeed, the observed *Mbtps1* exon 2 ablation seen in neutrophils from all six Cko mice that we analyzed indicate a causal role of S1P to the neutrophil phenotype. This indicates a role for S1P in the maintenance of the hematopoietic lineage.

**Fig. 10.**
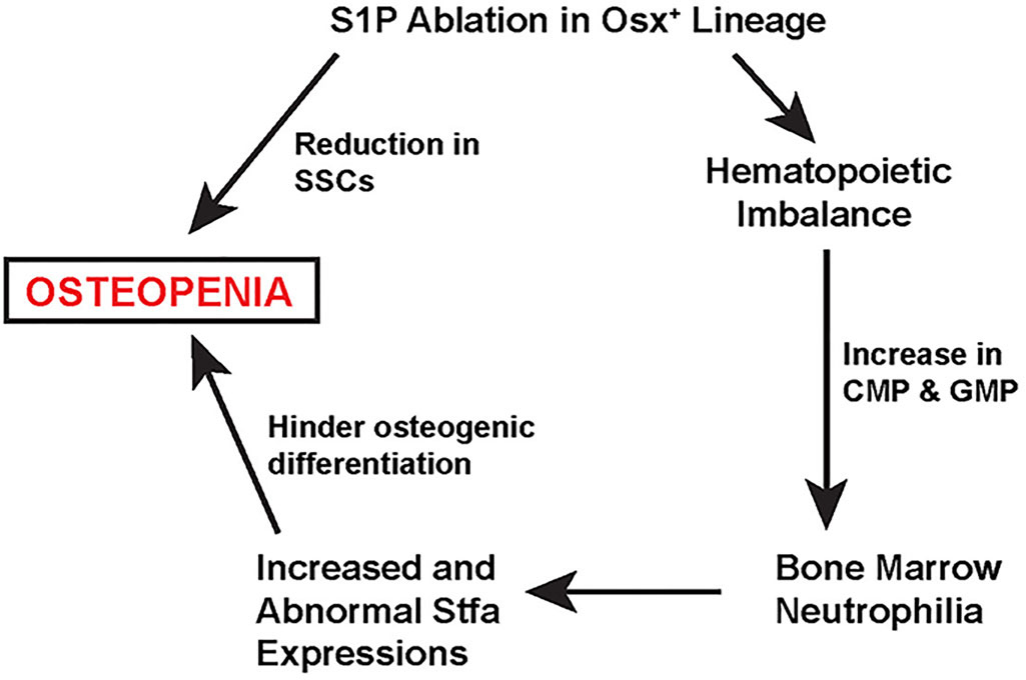
**A model showing the twofold detrimental effect on bone development due to S1P ablation in the Osx-lineage (see text for description).**

Thus, S1P has a pivotal role in maintaining the regenerative capacity of the bone marrow and balancing osteoblastogenesis with hematopoiesis. Fig. 10 shows a model delineating the contribution of S1P in maintaining the reciprocal relationship between osteogenesis and hematopoiesis. The resulting osteopenia on S1P ablation in the Osx-lineage could happen by two different pathways, acting simultaneously and additively to adversely affect bone development. S1P ablation results in decrease of SSCs that reduces osteoblastogenesis with decreased bone formation rate and mineral apposition rate (Patra et al., 2018). The current study demonstrated that S1P also balances hematopoietic progenitor synthesis. Its ablation results in the increased production of CMPs and GMPs and a special population of neutrophils that over-express many different kinds of stefin proteins. Stefins hinder osteogenic differentiation and thus exacerbate the decrease in SSCs to further reduce bone development. An investigation of the molecular pathway(s) that S1P modulates through its protease activity will allow for an in-depth understanding of the interplay between S1P and the hematopoietic and skeletal progenitors in the bone marrow and the regulatory events that help maintain skeletal homeostasis. It may also help us understand the molecular underpinnings of hematopoietic abnormalities with myeloid neoplasms such as myelodysplastic syndromes and acute myeloid leukemia (van de Loosdrecht et al., 2008).

## MATERIALS AND METHODS

### Ethics statement

All mouse procedures were performed in accordance with the NIH's Guide for the Care and Use of Laboratory Animals using vertebrate animals/ethics protocols reviewed and approved by the Animal Studies Committee at Washington University School of Medicine.

### S1P ablation in the Osx-lineage in mice

Mice with S1P ablation in the Osx-lineage were generated as described previously (Patra et al., 2018). Briefly, S1P*^f/f^* mice (mice homozygous for the floxed exon 2 of *Mbtps1*) (Yang et al., 2001) were bred with *Osx1-GFP::Cre* (Rodda and McMahon, 2006) mice in C57BL/6J strain to produce S1P*^+/f^*;*Osx-Cre* mice (mice heterozygous for S1P*^flox^* allele with *Osx-Cre* transgene). The heterozygous S1P*^+/f^*;*Osx-Cre* (S1P*^+/f-Osx^*) mice were bred with S1P*^f/f^* mice to generate mice with homozygous deletion of S1P (S1P*^f/f^*;*Osx-Cre* or S1P*^cko-Osx^* or Cko) in the Osx-lineage. Genotypes were verified by PCR analysis of tail-derived DNA. Male and female mice showed identical phenotypes. *Osx-Cre* mice (Huang and Olsen, 2015) were used as controls in the previous study to confirm that the mutant phenotypes were caused by S1P ablation (Patra et al., 2018). Therefore, *Osx-Cre* mice have not been used anymore in this study.

### μCT analysis of scoliotic spines

For μCT analyses of scoliotic spines, mice were skinned, eviscerated, fixed in 10% neutral buffered formalin for 24–48 h, washed and stored in 70% ethanol. Spines were scanned in a microCT 40 scanner (Scanco Medical AG, Switzerland) at high resolution and tube settings of 70 kV peak of energy, 114 microamperes of current with an integration time of 300 ms. Scanned data were converted to DICOM file format using scanco's onboard software. The generated DICOM files were used in reconstructing three-dimensional (3D) images using Dragonfly (Object Research Systems Inc., Canada) with 8 Color Realistic 3D lookup table. The reconstructed 3D images of bone were analyzed for their morphological differences in various vertebral regions.

### Proteomics of BMCs

#### Preparation of RBC-free BMCs

Bone marrow was harvested from the limbs of WT and Cko mice. After thorough removal of the skin and muscle, the limbs were crushed in a mortar and pestle in the presence of PBS (no Ca^2+^, no Mg^2+^) and strained through a 70 µm filter and the filtrate stored on ice. Crushed bones and leftover tissue in the filter were digested with 0.25% collagenase (Stem Cell Technologies, # 07902) at 37°C for 15 min, strained through a 70 µm filter and pooled with the filtrate from above. The filtrate/cells were washed three times with cold PBS and treated with RBC lysis buffer (Sigma-Aldrich, R7757) for 5 min. Following washings with cold PBS to remove the lysed RBCs and hemoglobin, cells were suspended in buffer (PBS with 0.1% bovine serum albumin), filtered through a 40 µm filter and counted.

#### Preparation of BMC lysate

RBC-free BMCs were prepared from the hind limbs of 21–23 day old WT and S1P^*cko-Osx*^ mice as above and lysed in 500 µl of T-PER (a tissue protein extraction reagent, Thermo Fisher Scientific, product # 78510) containing Complete Mini EDTA-free protease inhibitor cocktail (Roche) and phosphatase inhibitor cocktail (Roche). After centrifugation (10,000 rpm for 5 min at 4°C) to remove cell debris, the protein lysate was stored at −80°C until processed. For tryptic analysis by LC/MS/MS the lysate was precipitated by addition of ice cold acetone (final concentration 80%) and overnight incubation at −20°C to remove trypsin inhibitors and other small molecules. After centrifugation at 15,000× ***g*** for 10 min, protein pellet was washed twice with ice cold 100% acetone and solubilized in SDT buffer (4% SDS, 100 mM DTT, 100 mM Tris-HCl pH 7.6) using AFA sonication (Covaris S220X). Protein concentration was determined by BCA (Pierce, cat# 23225). Equal portions of lysate from each sample were pooled to make a common reference pool. The reference pool and experimental samples were then subjected to tryptic digestion and mass spec analysis after TMT labeling.

#### Tryptic digestion and TMT labeling

All samples were digested as previously described (Wiśniewski et al., 2009) with a few modifications (Erde et al., 2014). Briefly, 50 µg of protein in 50 µl SDT buffer was mixed with 400 µl 100 mM Tris-HCl buffer, pH 8.5 containing 8 M urea and transferred on top of a 30K filter (Millipore, part# MRCF0R030) and spun in a microcentrifuge at 10,000× ***g*** for 15 min. An additional 400 µl of 100 mM Tris-HCl buffer, pH 8.5 containing 8 M urea was added on top of the filter and the filter was spun at 10,000× ***g*** for 15 min in a microcentrifuge. The flow through was discarded and the proteins were alkylated by addition of 100 µl of 50 mM iodoacetamide on top of the filter, mixing at 550 rpm and incubation at RT for 20 min in the dark. The filter was spun at 10,000× ***g*** for 10 min and the flow through discarded. Unreacted iodoacetamide was washed through the filter with application of 2×200 µl of 100 mM Tris-HCl buffer, pH 8.5 containing 8 M urea with spinning at 10,000× ***g*** for 10 min after each addition. The urea buffer was exchanged into 100 mM ammonium bicarbonate buffer, pH 8 with two additions of 200 µl each and spinning after each addition. The filters were transferred to a new collection tube and 100 µl of 0.05 µg/µl Trypsin in 100 mM ammonium bicarbonate buffer, pH7.5 was added to each filter. Samples were digested overnight at 37°C in a humidified chamber. After overnight incubation, an additional 1 µg of Trypsin was added to the top of the filter for a 4 h digest at 37°C in a humidified chamber. The filters were spun at 10,000× ***g*** for 15 min to collect the peptides in the flow through. The filter was washed with 50 µl 0.1 M Tris-HCl buffer (pH 8.0) and the wash was collected with the peptides. Residual detergent was removed by ethyl acetate transfer, followed by acidification to a final concentration of 1% trifluoroacetic acid (TFA) in preparation for desalting. Peptides were desalted using micro-tips (C4, BIOMEKNT3C04 and porous graphite carbon, BIOMETNT3CAR) (Glygen) on a Beckman robot (Biomek NX), as previously described (Chen et al., 2012).

The peptides were eluted with 60% acetonitrile in 0.1% TFA and pooled for drying in a Speed-Vac after adding TFA to 5%. The peptides were dissolved in 20 µl of 1% acetonitrile in water. An aliquot (10%) was removed for quantification using Pierce Quantitative Fluorometric Peptide Assay kit (Thermo Fisher Scientific, Cat. No. 23290). The remainder of the peptides from each sample (1.5–1.8 µg) were transferred into a new tube, dried in the Speed-Vac and then dissolved in 12 µl of 100 mM HEPES buffer (pH 8.0). The samples were labeled with ten-plex tandem mass tag reagents (TMT10) (Thermo Fisher Scientific) according to the manufacturer's protocol. Labeled samples were pooled, dried, and resuspended in 250 µl aqueous 1% formic acid (FA). The TMT10 labeled samples were desalted as described above for unlabeled peptides. The eluates were transferred to autosampler vials (Sun-Sri, Cat. No. 200046), dried and stored at −80°C until LC/MS/MS analysis.

#### *nano*-LC/MS/MS analysis

2.5 µl of samples in 1% FA were loaded onto a 75 µm i.d.×50 cm Acclaim^®^ PepMap 100 C18 RSLC column (Thermo Fisher Scientific) on an EASY *nano*LC (Thermo Fisher Scientific) at a constant pressure of 700 bar at 100% buffer A (aqueous 0.1% FA). Prior to sample loading the column was equilibrated to 100% buffer A for a total of 20 µl at 700 bar pressure. Peptide chromatography was initiated with mobile phase A (1% FA) containing 5% buffer B (100% acetonitrile, 1% FA) for 1 min, then increased to 15% buffer B over 108 min, to 25% buffer B over 87 min, to 35% buffer B in 40 min, to 70% buffer B in 6 min, to 95% buffer B over 2 min and held at 95% B for 18 min, with a flow rate of 300 nl/min. The data was acquired in data-dependent acquisition (DDA) mode. The full-scan mass spectra was acquired with the Orbitrap mass analyzer with a scan range of *m/z*=375 to 1500 and a mass resolving power set to 70,000. Twelve data-dependent high-energy collisional dissociations were performed with a mass resolving power set to 35,000, a fixed lower value of *m/z* 100, an isolation width of 1.2 Da, and a normalized collision energy setting of 32. The maximum injection time was 60 ms for parent-ion analysis and 120 ms for product-ion analysis. The target ions that were selected for MS/MS were dynamically excluded for 20 s. The automatic gain control (AGC) was set at a target value of 3e6 ions for full MS scans and 1e5 ions for MS2. Peptide ions with charge states of one or >7 were excluded for CID acquisition.

#### Identification of proteins

MS raw data were converted to peak lists using Proteome Discoverer (version 2.1.0.81, Thermo Fischer Scientific) with the integration of reporter-ion intensities of TMT10 at a mass tolerance of ±3.15 mDa (Werner et al., 2014). MS/MS spectra with charges +2, +3 and +4 were analyzed using Mascot search engine (Perkins et al., 1999) (Matrix Science, London, UK; version 2.5.1). Mascot was set up to search against a Reference Sequence (RefSeq version July, 2013) database of mouse (24,821 entries), and common contaminant proteins (cRAP, version 1.0 January 1st, 2012, 116 entries), assuming the digestion enzyme was trypsin/P with a maximum of four missed cleavages allowed. The searches were performed with a fragment ion mass tolerance of 0.02 Da and a parent ion tolerance of 20 ppm. Carbamidomethylation of cysteine was specified in Mascot as a fixed modification. Deamidation of asparagine, formation of pyro-glutamic acid from N-terminal glutamine, acetylation of protein N-terminus, oxidation of methionine, and pyro-carbamidomethylation of N-terminal cysteine were specified as variable modifications. Peptide spectrum matches (PSM) were filtered at 1% FDR by searching against a reversed database and the ascribed peptide identities were accepted. The uniqueness of peptide sequences among the database entries was determined using the principal of parsimony. Protein identities were inferred using a greedy set cover algorithm by Mascot and the identities containing ≥2 Occam's razor peptides were accepted (Koskinen et al., 2011).

#### Protein relative quantification

The processing, quality assurance and analysis of TMT data were performed with proteoQ (development version 1.0.1.2, https://github.com/qzhang503/proteoQ), a tool developed with the tidyverse approach (https://CRAN.R-project.org/package=tidyverse) (Wickham, 2019a, 2019b) under the free software environment for statistical computing and graphics, R (https://www.R-project.org/) and RStudio (http://www.rstudio.com/) (R Studio Team, 2016). Briefly, reporter-ion intensities under 10-plex TMT channels were first obtained from Mascot, followed by the removals of PSM entries from shared peptides or with intensity values lower than 1E3. Intensity of PSMs were converted to logarithmic ratios at base two, in relative to the average intensity of reference samples within a 10-plex TMT. Under each TMT channel, Dixon's outlier removals were carried out recursively for peptides with greater than two identifying PSMs. The median of the ratios of PSM that can be assigned to the same peptide was taken to represent the ratios of the incumbent peptide. The median of the ratios of peptides were taken to represent the ratios of the incumbent protein. To align protein ratios under different TMT channels, likelihood functions were first estimated for the log-ratios of proteins using finite mixture modelling, assuming three-component Gaussian mixtures (R package: mixtools:: normalmixEM) (Benaglia et al., 2009). The ratio distributions were then aligned in that the maximum likelihood of the log-ratios are centered at zero for each sample. Scaling normalization was performed to standardize the log-ratios of proteins across samples by dividing the log-ratios with respective to the standard deviation of each sample. To discount the influence of outliers from either log-ratios or reporter-ion intensities, the upper and the lower 5% of log2-ratios and the log2-ratios that are associated with the upper and the lower 5% of reporter-ion intensity were removed from the calculations of standard deviation.

#### Informatic and statistical analysis

Metric multidimensional scaling (MDS) and principal component analysis (PCA) of protein log2-ratios was performed with the base R function stats::cmdscale and stats:prcomp, respectively. Heat-map visualization of protein log2-ratios was performed with pheatmap (https://CRAN.R-project.org/package=pheatmap) (Kolde, 2019) within proteoQ. Linear modeling were performed using the contrast fit approach in limma (Ritchie et al., 2015) to assess the statistical significance in protein abundance differences between indicated groups of contrasts. Adjustments of *P*-values for multiple comparison were performed with Benjamini-Hochberg (BH) correction.

### Single cell RNA-seq analysis

#### Harvesting of single cells (Sc) for RNA-Seq

For Sc-FACS-Seq analysis of GFP^+^ cells, BMCs were prepared from the hind limbs of P21 Cko mice (see above) and GFP^+^ cells were FACS sorted using a BD FACSAria II (Becton Dickinson) FACS sorter into a sterile 96-well plate at a density of one cell/well containing 10X lysis buffer (Clontech, # 635013) with Protector RNAse inhibitor (Roche). The plates were frozen immediately on dry-ice and stored at −80°C until ready to process for RNA-Seq. Two 96 well plates of GFP^+^ sorted single cells (GFP-Cko1, GFP-Cko2) were carried forward for analysis with each plate receiving cells sorted from a pool of BMCs made from two Cko mice. Thus, a total of four Cko mice were analyzed. For RNA-Seq analysis of skeletal stem cells (SSCs) defined as CD45^−^, Ter-119^−^, CD31^−^, CD105^+^ cells (Patra et al., 2018), 10^6^ cells from WT and Cko mice were stained as reported previously with fluorescent-tagged antibodies to CD45 (PE-Cy7) (BD Pharmingen, 552848), Ter-119 (PE-Cy7) (eBioscience, 25-5921), CD31 (BV421) (BD Pharmingen, 563356), and CD105 (Alexa-fluor 647) (BD Pharmingen, 562761), washed to remove unincorporated antibodies, and SSCs were FACS sorted at a density of one cell/well into a sterile 96-well plate containing lysis buffer. A total of two WT (SSC-WT1, SSC-WT2) and two Cko (SSC-Cko1, SSC-Cko2) mice were analyzed by RNA-Seq.

#### Sc-RNA-Seq library preparation from FACS sorted cells

Following FACS sorting of single cells into lysis buffer, mRNA was primed in each well with an oligo dT primer that incorporates a unique 10 base pair (bp) barcode and Takara's SMART sequence to the 3′ ends and reverse transcribed to yield cDNA using SMARTscribe RT enzyme (Takara) and a template switching oligo to incorporate the SMART sequence to the 5′ ends. These cDNAs from each well were combined and purified with Ampure XP beads and amplified for 16 cycles using an oligo corresponding to the SMART sequence. Full-length cDNAs were sheared using Covaris E220 instrument with peak incident power 18, 20% duty factor, 50 cycles/burst for 2 min. The cDNAs were blunt-ended, an A base added to the 3′ ends, and Illumina sequencing adapters ligated to these ends. Ligated fragments were then amplified for 16 cycles using standard i7 indexing primer and P5 primer that binds only to the 3′ ends of the cDNAs containing SMART sequences and barcodes. Fragments were sequenced on an Illumina HiSeq-2500 using paired reads extending 26 bases for read one to read cell barcodes, and 98 bases for read two for mRNA data. Each plate was represented by a unique index and each well is represented by unique barcode.

#### Sc-RNA-Seq library preparation using the 10× Genomics platform

For single cell digital expression profiling of several thousand BMCs in a microfluidic platform analysis by 10× Genomics (www.10xgenomics.com), BMCs were prepared from one P21 WT and S1P*^cko-Osx^* mice as above and cells were processed using the Chromium Controller and Chromium Single Cell 3′ Library & Gel Bead Kit v2 and the manufacturer's recommended protocol to make unique barcoded single cell RNA sequencing libraries. The libraries were sequenced on an Illumina HiSeq2500 using paired-end 26×98 bp as the sequencing mode and to a depth of 50,000 reads/cell targeting approximately 170 million reads per genotype.

#### RNA-Seq library analysis, quality control and processing

Sequencing reads from FACS-Seq were aligned to the Ensembl (release 76) top-level assembly using STAR (version 2.0.4b) as above. Gene counts were derived from the number of uniquely aligned unambiguous reads by Subread:featureCount version 1.4.5. Isoform estimated counts were produced by Sailfish version 0.6.3. Gene and isoform counts were further transformed into counts-per-million (CPM), log 2 CPM with a prior count of 2 (moderated log2CPM), and RPKM with custom Rscripts. Sequencing performance was assessed for total number of aligned reads, total number of uniquely aligned reads, genes and transcripts detected, ribosomal fraction, known junction saturation and read distribution over known gene models with RSeQC version 2.3. For 10x Genomics reads, alignments and gene quantification were done with 10x Cellranger software.

All gene-level counts were then imported into R package RaceID where genes and samples were pre-filtered to only include genes present in at least five samples that have a minimum of at least 3000 genes detected with a minimum expression of 1 count to a maximum of the median max of counts across all samples with no downsampling. All reads from autosomal and mitochondrial ribosomal RNA were excluded from further analysis. These pre-filtered genes and samples were then clustered with k-means hierarchical clustering as described by RaceID, but no outliers were excluded or reclustered. The resulting clustered samples were then examined for genes that were significantly expressed in each cluster versus all other clusters by RaceID's negative binomial modeling and the gene lists were further examined with the R package clusterProfiler to identify what GO biological processes and molecular function terms were best associated with each cluster via hypergeometric enrichment tests and the top five terms found were further illustrated with category network plots. The gene-level counts were also imported into the R package Seurat where the data was filtered to remove low expressing genes, normalized, and then adjusted to account for the confounding effects of mitochondrial and ribosomal genes via Seurat's regression model. The principle components were examined and clusters of samples were generated. The genes characterizing each cluster versus the others by Seurat were then interrogated by clusterProfiler as was done previously with the RaceID results.

### FACS analysis for neutrophils

For FACS analysis of the neutrophil population and stefin-expressing neutrophil subset, BMCs were prepared as described above from P21 WT and S1P*^cko-Osx^* mice. Cells were counted and resuspended in FACS buffer (2% fetal bovine serum and 0.25 mM EDTA in PBS) and counted. The neutrophil population was determined by assaying for the CD45^+^ CD11b^+^ Ly6G^+^ population (Kim et al., 2017; Shi et al., 2011). For this, 5 million BMCs were stained with 5 µl each of PE-Cy7 conjugated rat anti-mouse CD45 (clone 30-F11, BD Pharmingen, 552848), PerCP-Cy5.5 conjugated rat anti-CD11b (clone M1/70, BD Pharmingen, 550993), FITC conjugated rat anti-mouse Ly6G (clone 1A8, BD Pharmingen, 581460), Super Bright 436 conjugated rat anti-mouse Ly6C (eBioscience, 62-5932-82) antibodies and the fixable viability stain (FVS) 575 V (BD Horizon, 565694) for 50 min. Unstained cells and Fluorescence Minus One antibody (FMO) for each antibody, and isotype controls were also included. After washing with flow cytometer staining buffer (eBioscience, 4222-26), the cells were fixed with Fixation Buffer (eBioscience, 88-8824) following the manufacturer's recommended protocol and titrated in a BD LSR II (BD Biosciences) flow cytometer and analyzed by FlowJo (www.flowjo.com). To determine the neutrophil population by FlowJo (Fig. S7), single cells and live cells were gated and the PE-Cy7-CD45^+^ myeloid population was selected. Within the CD45^+^ population, the PerCP-Cy5.5-CD11b^+^ and FITC-Ly6G^+^ double-positive population was selected to define the triple-positive CD45^+^ CD11b^+^ Ly6G^+^ neutrophil population; additionally, the PerCP-Cy5.5-CD11b^+^ and Super Bright 436-Ly6C^+^ double-positive population was also analyzed. Unstained WT cells and PE-Cy7-conjugated rat IgG2b kappa isotype (clone RTK4530, BioLegend, # 400617) stained controls showed insignificant non-specific staining in the gated neutrophil populations in test samples. FMO controls were used to determine the boundaries of each gate. WT and Cko mice from several different litters were tested and their numbers are shown in figure legends.

To determine the stefin-expressing neutrophil population, we used rabbit anti-human stefin A antibody (My BioSource, # MBS852720) in combination with the CD45, CD11b, Ly6G antibodies. As stefin is an intracellular protein, the following protocol was performed. After staining with CD45, CD11b and Ly6G antibodies, the cells were washed with Flow Cytometry staining buffer and fixed and permeabilized using an intracellular fixation and permeabilization buffer set (eBioscience, # 88-8824-00) and the manufacturer's recommended protocol. Next, after blocking the cells with 10% normal goat serum for 10 min, the cells were incubated with 5 µl of the rabbit anti-human stefin A antibody for 30 min at room temperature (RT). After washing to remove excess Ab, cells were treated with 5 µl of Alexa Fluor 594-conjugated goat anti-rabbit IgG secondary antibody (Invitrogen, A-11012) for 30 min at RT to detect the presence of stefin. Following washings, the cells were titrated in a LSR II flow cytometer and analyzed by FlowJo as above to assay for the stefin^+^ cells within the CD45^+^ CD11b^+^ Ly6G^+^ population. An FMO for the stefin A antibody was used to define the stefin-positive gate.

### FACS analysis for HSCs

For FACS analysis of HSCs, BMCs were prepared as above and resuspended in PBS. Cells were counted and 10 million cells were used to analyze the HSCs population for both WT and Cko mice. In addition to FVS575V stain, cells were stained with the following antibodies for 50 min: eFluor 450 conjugated mouse hematopoietic lineage cocktail (Thermo Fisher Scientific, # 88-7772-72), Alexa Fluor 700 conjugated rat anti-mouse Sca1 (Ly6A/E) (Thermo Fisher Scientific, # 56-5981-82), Super Bright 702 conjugated rat anti-mouse c-Kit (clone 2B8) (Thermo Fisher Scientific, # 67-1171-82), PE-Cy7 conjugated rat anti-mouse CD16/32 (Thermo Fisher Scientific, # 25-0161-82), FITC conjugated rat anti-mouse CD34 (Thermo Fisher Scientific, # 11-0341-85), PE conjugated rat anti-mouse CD150 (Thermo Fisher Scientific, # 12-1502-82), and Super Bright 600 conjugated rat anti-mouse CD48 (Thermo Fisher Scientific, # 63-0481-82). Unstained cells, FMO control for each antibody, and isotype controls were included for gating purposes in FlowJo. As above, cells were washed with flow cytometer staining buffer, fixed and titrated in a BD LSR II FACS machine. In FlowJo, after gating for single and live cells, the LIN^−^ gate containing HSCs was determined. Within the LIN^−^ population, the Sca1^+^ c-Kit^+^ double-positive LSK progenitor cells were determined and this population was gated further to determine CD48^−^ CD150^+^ LSK SLAM HSCs. The single positive Sca1^−^ c-Kit^+^ population was analyzed to determine the CD34^−^ CD16/32^+^ population, the CD34^+^ CD16/32^+^ GMP (granulocyte macrophage progenitor), the CD34^+^ CD16/32^−^ CMP (common myeloid progenitor), and the CD34^−^ CD16/32^−^ MEP (megakaryocyte-erythrocyte progenitor) progenitors (Akashi et al., 2000; Challen et al., 2009; Kondo et al., 1997; Seita and Weissman, 2010). WT and Cko mice from several different litters were tested and their numbers are shown in figure legends.

### *In vitro* osteogenic and adipogenic differentiation assays

Bone marrow was harvested from the hind limbs of P21 WT (S1P*^f/f^*) mice and the resulting cell population obtained after lysis of RBCs were cultured in a single well of a six-well plate in α-MEM with 20% FBS and 2% penicillin/streptomycin until BMSCs were confluent. Once confluent, cells were passaged into a 6 cm dish and cultured until confluent. The cells were then cultured in two wells of a six-well plate, grown until confluent at which point osteogenic differentiation media (α-MEM with 10% FBS, 2% penicillin/streptomycin, 10 mM β-glycerophosphate, 50 μg/ml of ascorbic acid), or adipogenic differentiation medium [MesenCult™ Adipogenic Medium (STEMCELL Technologies)] and recombinant N-terminal 6-histidine tagged (His6) stefin 1 or stefin 2 proteins were added and cultured. Cells were incubated at 37°C in normoxia conditions with a change in medium every 3 days. Osteogenic differentiation was assessed by silver staining of the mineralized matrix by the von Kossa method after 14 days, or 7 days for qPCR analysis of osteogenic markers, or 3 days for immunocytochemistry of His6-Stfa1/2. Recombinant His6-Stfa1 and His6-Stfa2 were custom ordered from My BioSource. Storage buffer (10 mM Tris-HCl, 1 mM EDTA, pH 8.0, 50% glycerol) was added as control (CTRL) to the same volume as the added recombinant proteins. qPCR analysis of osteogenic differentiation markers using SYBR Green primer sets, 2X SYBR Green mix (Life Technologies/Applied Biosystems) using standard protocols and the comparative C_T_ method was performed as previously described (Patra et al., 2018). Adipogenic differentiation was analyzed by staining for lipid droplets by Oil Red O at day 7.

### IF detection of type II collagen, stefin, His6-tag, and endomucin

IF for type II collagen triple helical domain on formalin-fixed, paraffin embedded (FFPE) sections using the IIF antibody (provided by Dr M. Cremer) was done as described in our earlier studies (Zhu et al., 1999; Patra et al., 2007). IF for stefin was also performed on FFPE sections. 5 µm sections were rehydrated with PBS following normal procedures. Sections were treated with proteinase K (10 µg/ml) for 20 min at 37°C and washed in PBS. After incubation in 10% normal goat serum in PBS for 60 min, sections were incubated overnight with 1:400 diluted rabbit anti-human stefin A antibody (My BioSource, # MBS852720) in 1.5% goat serum at 4°C. The slides were washed with PBS and incubated with Alexa Fluor 594-tagged goat anti-rabbit secondary antibody for 60 min at RT, washed with PBS, stained with DAPI and analyzed by fluorescence microscopy in a Zeiss microscope.

For immunocytochemistry detection of His6-Stfa1/2, BMSCs were grown on cover slips in 12 well plates, and osteogenic differentiation medium and His6-Stfa1 or His6-Stfa2 (250 ng/ml) added when confluent. After 3 days, the wells were washed with PBS and cells on coverslips fixed with ice-cold methanol for 20 min. Following several washes, cells were treated with blocking buffer (1× PBS, 5% normal goat serum, 0.1% saponin) for 1 h at RT. Following this, cells were incubated overnight with anti-His6 antibody (1:100) (Abcam, ab9108) in antibody dilution buffer (1× PBS, 1% BSA, 0.1% saponin) at 4°C. Appropriate controls for the IF (no addition of primary antibody but addition of secondary antibody and use of CTRL storage buffer in place of stefin proteins) were also used to determine specific detection for the His6-tag. After several washes with PBS, cells were treated with Alexa Fluor 594-tagged goat anti-rabbit secondary antibody for 60 min at RT, washed with PBS, stained with DAPI and analyzed by fluorescence microscopy in a Zeiss microscope. IF for Endomucin was performed on P21 WT and Cko frozen hind limb tissues. 5 µm sections were thawed, fixed with cold acetone, and rehydrated with PBS. After incubation in 10% normal goat serum in PBS for 60 min, the sections were incubated overnight with rat monoclonal anti-Endomucin antibody (eBioV.7C7, eBioscience) in 1.5% goat serum at 4°C. The slides were washed with PBS and incubated with Alexa Fluor 594 tagged goat anti-rat secondary antibody for 60 min, washed with PBS, stained with DAPI and analyzed by fluorescence microscopy. Images were captured on an Eclipse E800 microscope (Nikon) using a 60×, 1.4 NA oil immersion objective and QImaging Retiga 2000R Fast 1394 camera and deconvolved using MetaMorph software (Molecular Devices) and Z-motor device (*Prior* Scientific™).

### Analysis of *Mbtps1* Exon 2 ablation in Cko neutrophils

BMCs were prepared from Cko mice as above and stained with CD45, Ly6G and CD11b antibodies (see above). After washing with PBS, the stained BMCs were suspended in FACS sorting buffer (2% normal goat serum, 1 mM EDTA pH 8.0, 1× PBS) and FACS sorted for CD45^+^ Ly6G^+^ CD11b^+^ triple-positive cells using BD FACSAria II. 20,000–30,000 cells were collected for the analysis. DNA was extracted from these cells and using primers (forward primer: 5′-GGGTGGGACATGACATCTGG-3′ located upstream of exon 2) and (reverse primer: 5′-GCTTTGGCTGTGAAGTATCCG-3′ from exon 3 of the *Mbtps1* gene), PCR was performed to analyze recombination of the floxed genomic exon 2 by *Osx-Cre* (Fig. S10). The PCR product of the expected size (∼1.5 kilobase) was extracted from gel and sequenced to confirm *Mbtps1* exon 2 ablation by standard Sanger sequencing protocols.

## Supplementary Material

Supplementary information
