# Supplementary material for: Site-1 protease ablation in the osterix-lineage in mice results in bone marrow neutrophilia and hematopoietic stem cell alterations
Source: Biol Open. 2020 Jun 23;9(6):bio052993. doi: 10.1242/bio.052993 (PMC7328000; doi:10.1242/bio.052993)
Supplement: Supplementary information [file biolopen-9-052993-s1.pdf]

SUPPLEMENTARY FIGURES

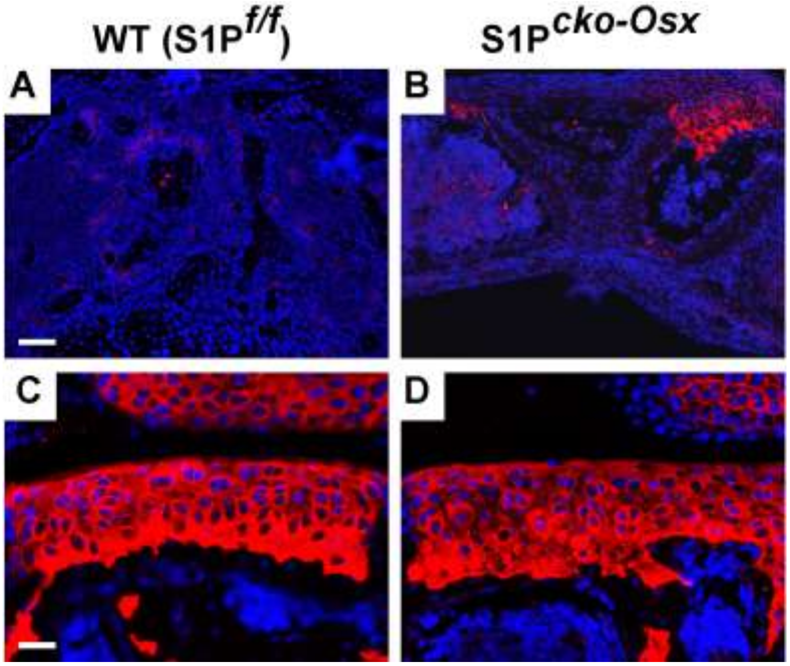

Figure S1 (Fig. S1). Immunofluorescence for type II collagen (red) in the posterior tip of the spinous process (in the region of SBO) in the Cko (B) and a corresponding region in the WT) (A), and in the synchondroses regions (C, D) in the WT and Cko C2 vertebrae. Blue= DAPI-stained nuclei. Bars: (A, B): 50 µm; (C, D): 25 µm.

Biology Open • Supplementary information

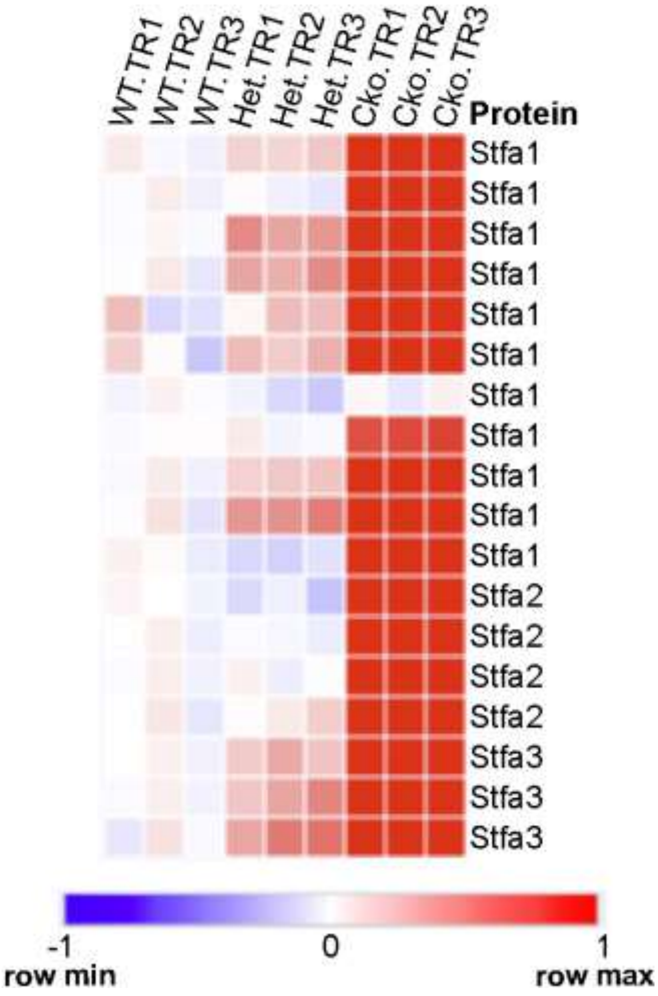

Figure S2 (Fig. S2). A Morpheus heatmap of the stefin (Stfa) family of proteins identified in a preliminary study in a single WT, Het and Cko BMC protein lysate. Each genotype has three technical repeats (TR1, TR2, TR3) and each row represents a different peptide sequence as identified by LC/MS/MS (also see Fig. 4).

Biology Open • Supplementary information

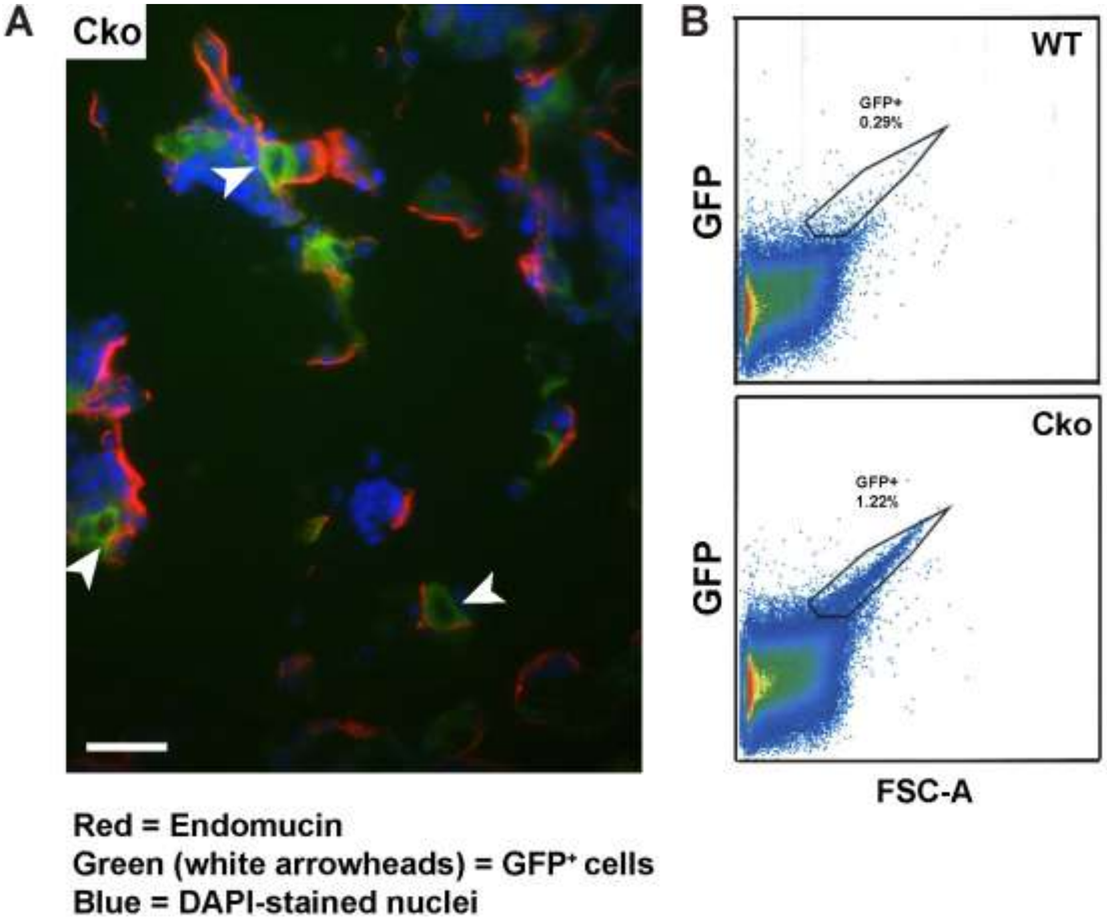

Figure S3 (Fig. S3). (A) Immunofluorescence for endomucin (red) in the femur of P21 Cko mouse showing the relative location of blood vessels to GFP$^+$ cells (white arrowheads). Blue=DAPI-stained nuclei. (B) FACS analysis of BMCs from P21 WT and Cko mice for GFP$^+$ cells; the relative amount of GFP$^+$ cells, compared to total BMCs analyzed, is shown for each. Bar: (A): 10 μm.

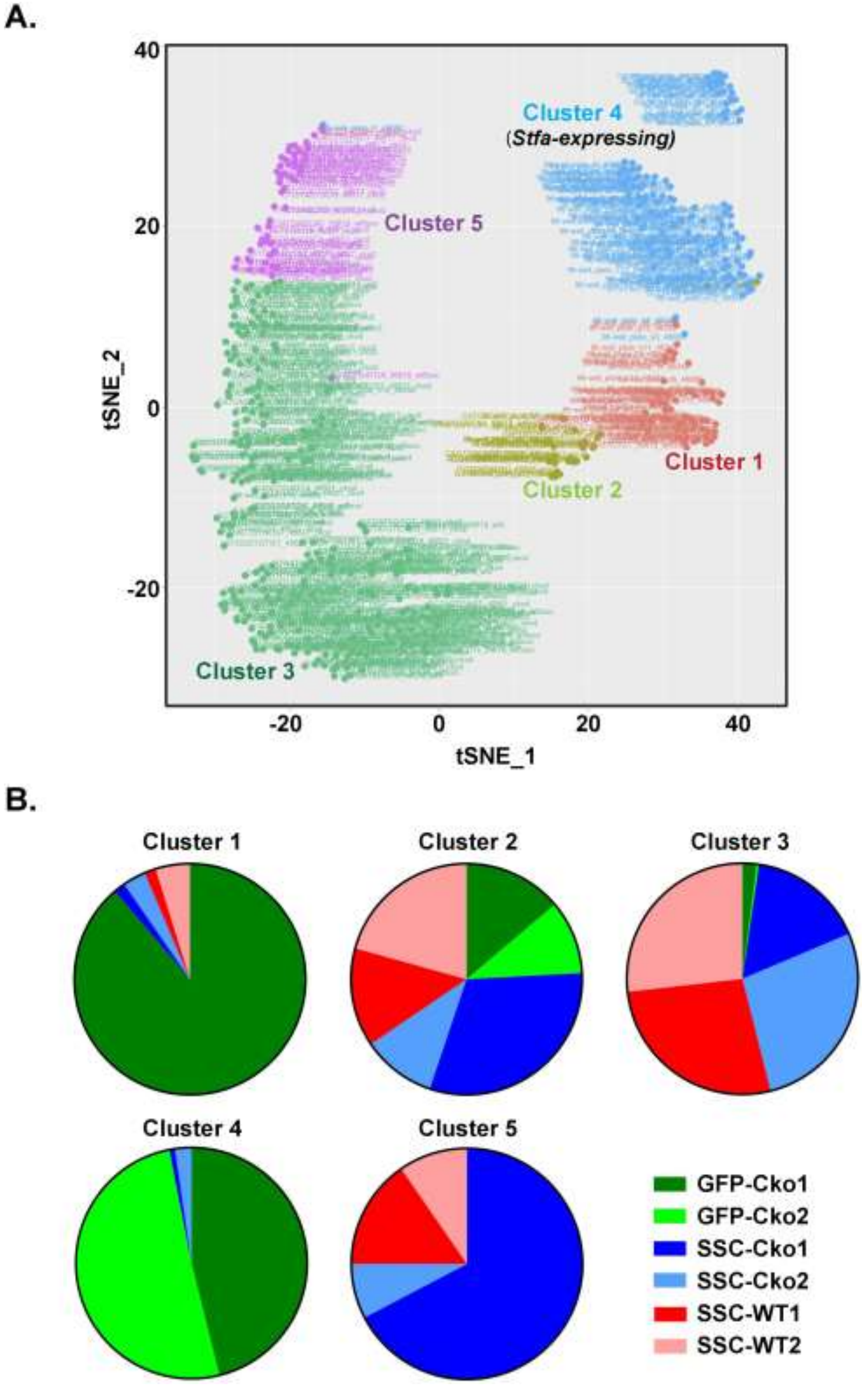

Biology Open • Supplementary information

Figure S4 (Fig. S4). (A) A tSNE plot from RaceID clustering analysis of Sc-RNA-Seq data of Cko-GFP^{+} cells and SSCs from WT and Cko, showing five distinct clusters (1-5). Like the Seurat analysis, the RaceID analysis is also based on Sc-RNA-Seq data from all six 96-well plates [two from GFP-sorted Cko cells (GFP-Cko1 & GFP-Cko2), two from WT SSCs (SSC-WT1 & SSC-WT2) and two from Cko SSCs (SSC-Cko1 & SSC-Cko2)]. The contribution of each plate to these clusters is shown color-coded in B.

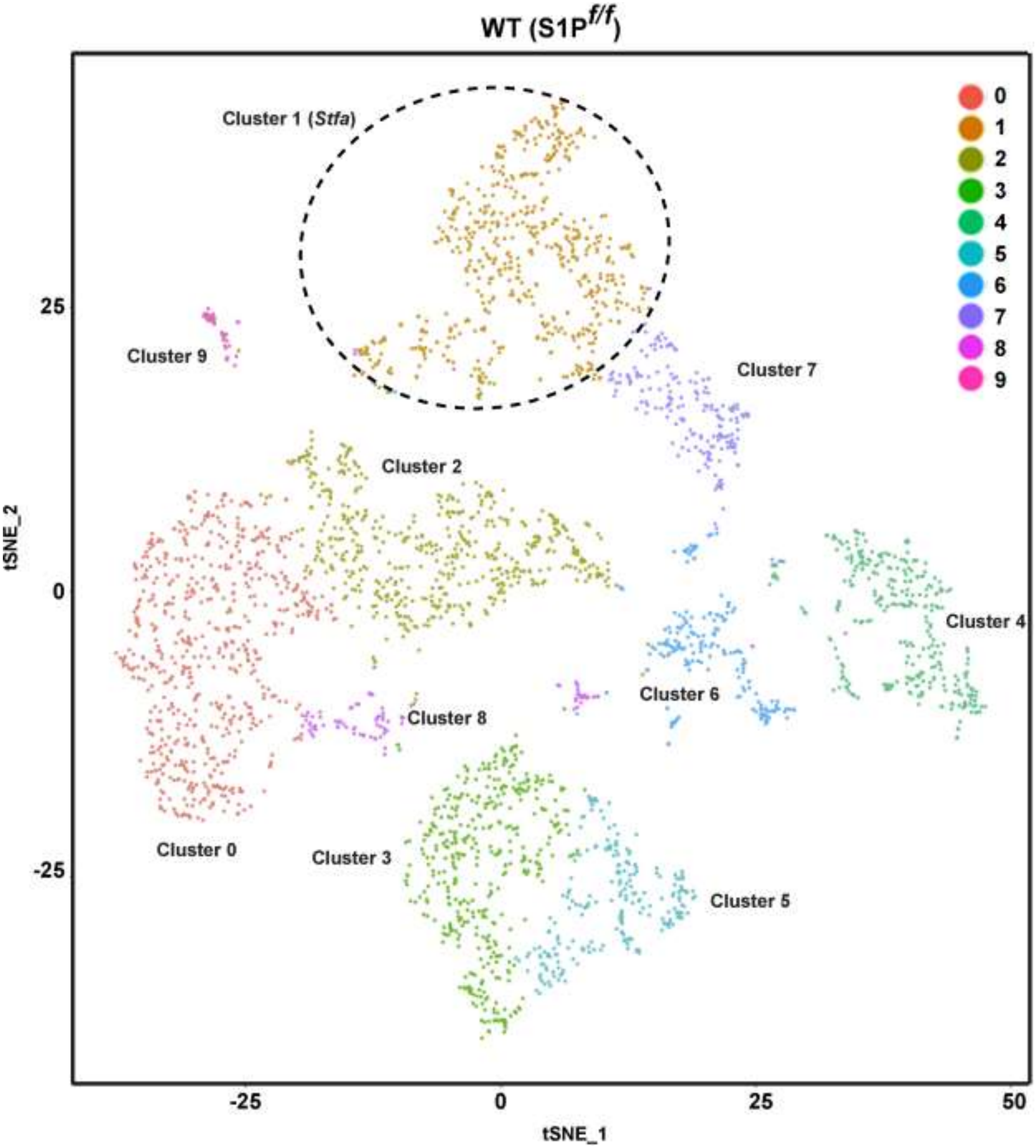

Figure S5 (Fig. S5). A tSNE plot derived from Seurat analysis of Sc-RNA-Seq data of WT BMCs using 10x Genomics, showing 10 distinct clusters. Compare with Fig. S6 and Tables 4, 5, 6 and S3. The single *Stfa*-expressing cluster 1 is shown marked by a dotted-ellipse.

Biology Open • Supplementary information

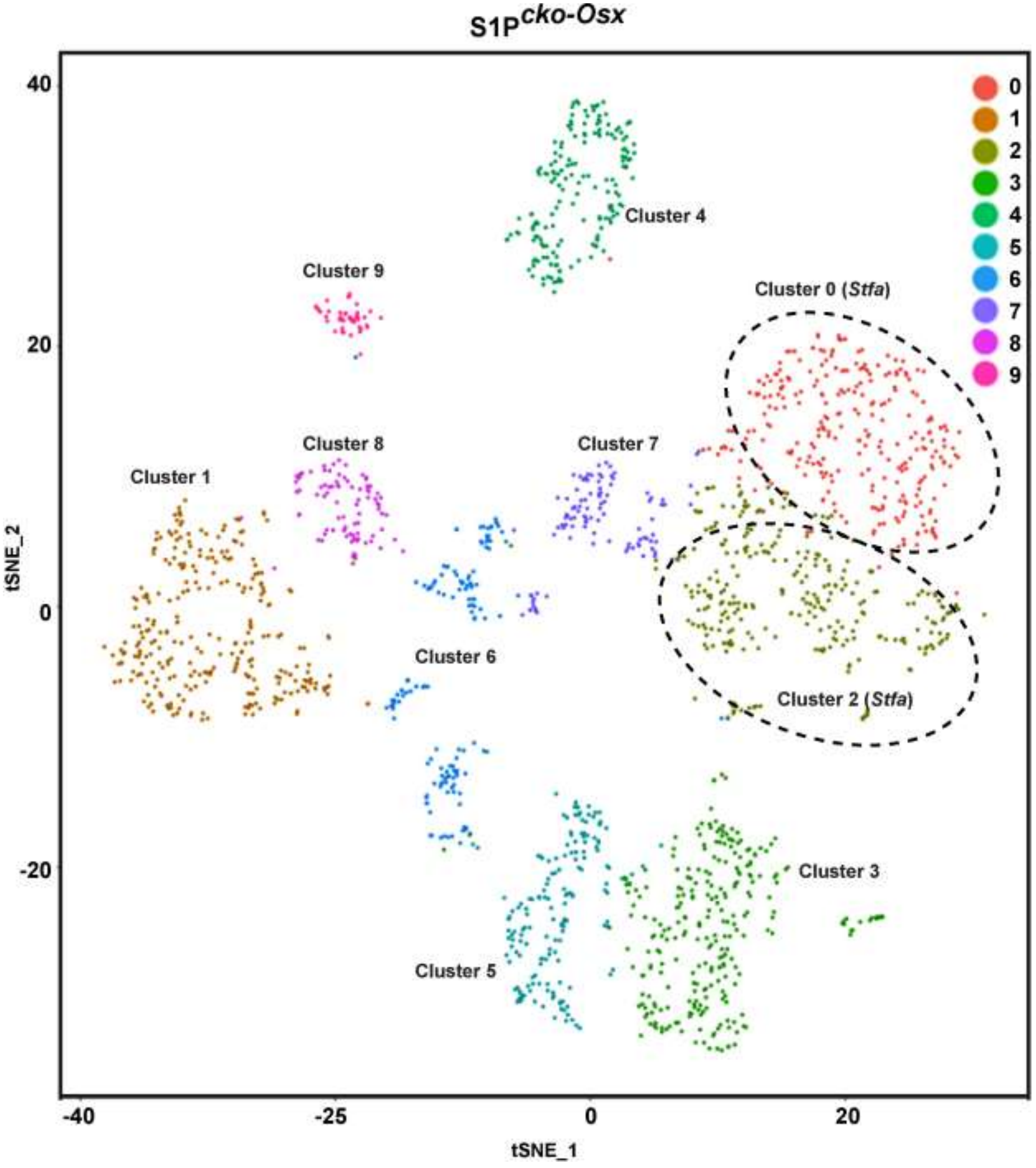

Figure S6 (Fig. S6). A tSNE plot derived from Seurat analysis of Sc-RNA-Seq data of Cko BMCs using 10x Genomics, showing 10 distinct clusters. Compare with Fig. S5 and Tables 4, 5, 6 and S3. The two *Stfa*-expressing clusters, cluster 0 and 2, are shown marked by a dotted-ellipse.

Biology Open • Supplementary information

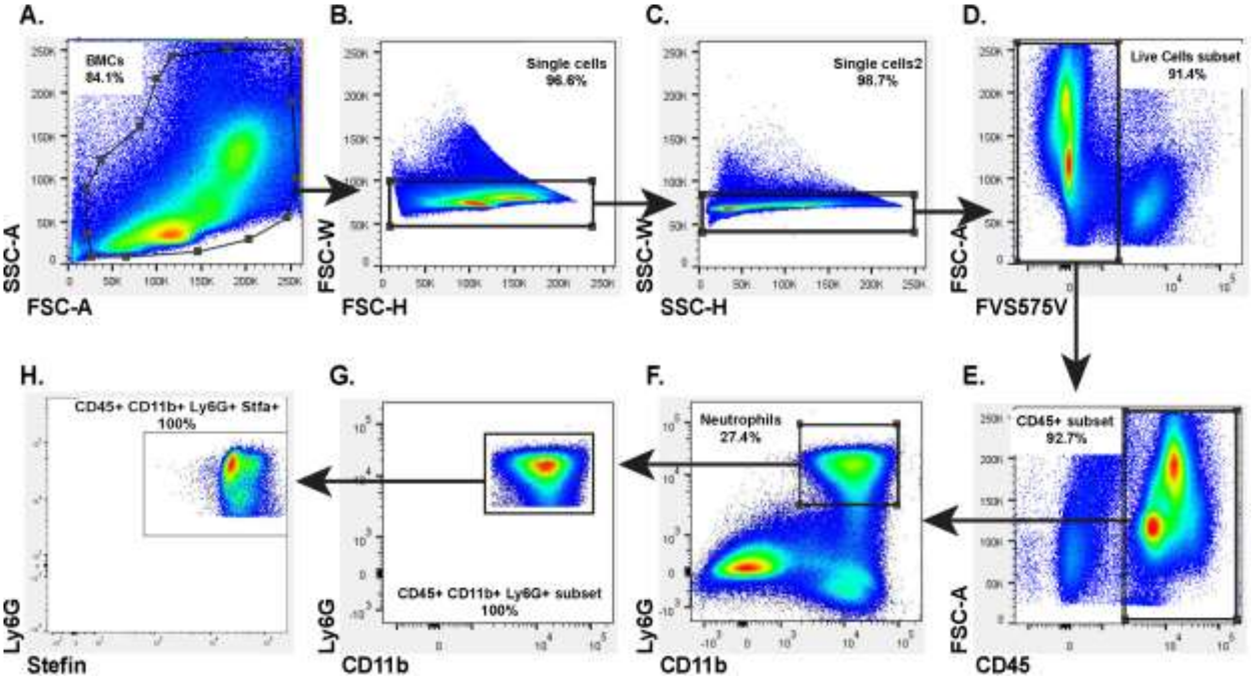

Figure S7 (Fig. S7). Figure shows the gating procedure used to quantitate the CD45$^+$ CD11b$^+$ Ly6G$^+$ triple-positive neutrophil population using the WT as an example. Arrows indicate the gate selected and carried forward for the next analysis. After gating for single cells (B, C) and live cells (D), the resulting population was gated for CD45$^+$ myeloid cells (E), followed by gating for Ly6G$^+$ CD11b$^+$ double-positive cells (F) within the CD45$^+$ population to give the triple positive neutrophil population. The neutrophils were gated further for stefin A (Stfa)-expressing cells (H) within this population. The percent figures are indicative of percent cell population selected in that gate (percent of parent) that had the desired marker, and carried forward to the next gate. Thus 27.4% of CD45$^+$ cells (which were gated in E) are also CD11b$^+$ Ly6G$^+$ positive (F). However, 100 percent of CD45$^+$ CD11b$^+$ Ly6G$^+$ triple-positive cells (G) are also positive for stefin (H, notice change in axis).

Biology Open • Supplementary information

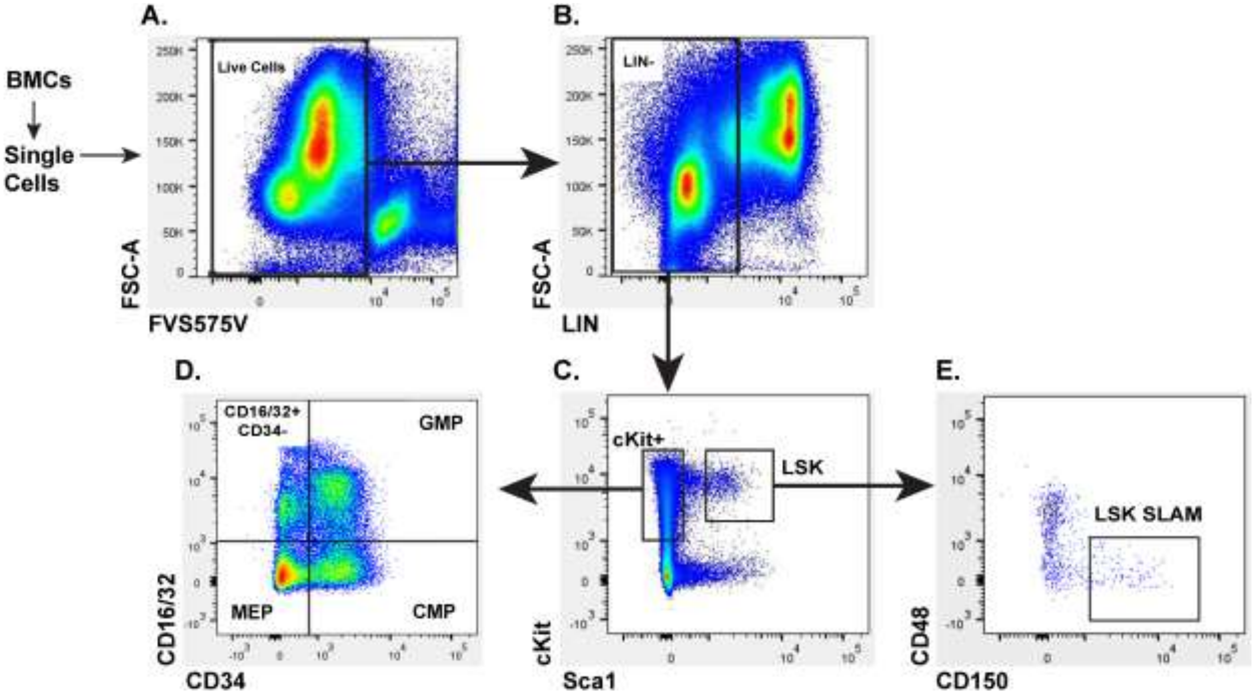

Figure S8 (Fig. S8). Figure shows the gating procedure used to titrate hematopoietic stem cells. Arrows indicate the gate selected and carried forward for the next analysis. After gating for single cells (as in Fig. S7) and live cells (A), the resulting population was gated for LIN$^-$ population (B). The LIN$^-$ population was gated for Sca1$^+$ c-Kit$^+$ double positive cells to give LSK cells (C) that were further gated for CD48$^-$ CD150$^+$ LSK SLAM cells (E). The LIN$^-$ population was also gated for Sca1$^-$ c-Kit$^+$ cells (C) that were further analyzed for MEP (CD34$^-$ C16/32$^-$), CMP (CD34$^+$ CD16/32$^-$), GMP (CD34$^+$ CD16/32$^+$) and the single positive CD16/32$^+$ cells (D).

Biology Open • Supplementary information

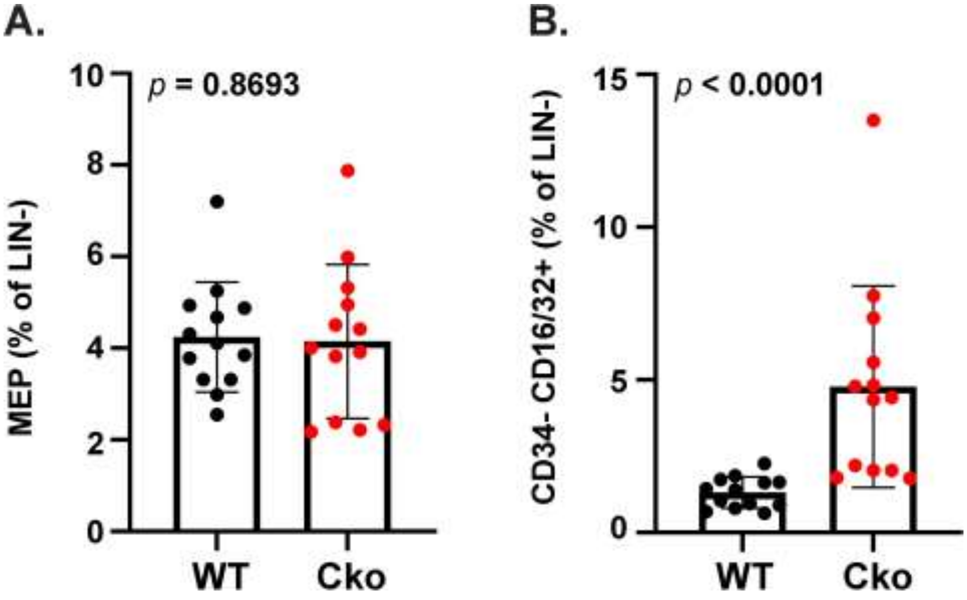

Figure S9 (Fig. S9). Analysis for MEP (A, % of LIN^-), and LIN^- $Sca1^-$ $c\text{-}Kit^+$ $CD34^-$ $CD16/32^+$ populations (B, % of LIN^-). The *p*-values for the comparison between WT and Cko were calculated using non-parametric t-test (two-tailed) with Mann Whitney correction from the analyses of WT ($N \geq 12$) and Cko ($N \geq 12$) mice from several different litters.

Biology Open • Supplementary information

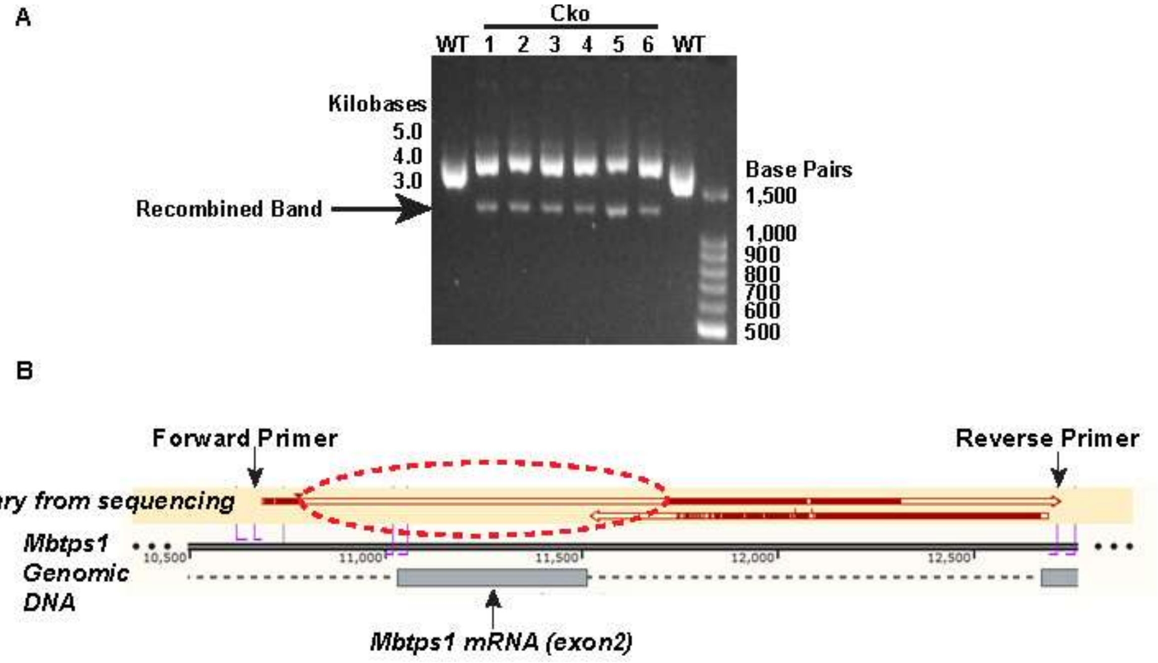

Figure S10 (Fig. S10). Analysis of *Mbtps1* (S1P) exon 2 ablation in Cko neutrophils. (A) Gel showing PCR products using forward and reverse primers (please see *Materials and Methods*) on DNA isolated from FACS-sorted CD45^{+} Ly6G^{+} CD11b^{+} triple positive cells. The arrow shows expected PCR product (~ 1.5 kb) from Cko neutrophils (*N*=6) due to recombination that removes the floxed *Mbtps1* exon 2. WT neutrophils were used as controls and do not show this product. (B) A graphical representation of the *Mbtps1* genomic sequence around exon2, with locations of forward and reverse primers and the structure of the mRNA. The panel above genomic DNA shows result from sequencing in each direction. The dotted red ellipse shows the boundary of genomic DNA missing in the Cko, after sequencing from both directions (arrows). The region deleted overlaps with *Mbtps1* exon2 sequence.

Biology Open • Supplementary information

Table S1. GO pathway analysis. Top 20 pathways identified by Gene Ontology (GO) that were changed in Cko BMCs as compared to the WT, based on proteomic analysis of RBC-free BMC lysates by LC/MS/MS.

	**GO Pathway**
**1**	Negative regulation of cytolysis
**2**	Cxcr3 chemokine receptor binding
**3**	Positive regulation of macrophage derived foam cell differentiation
**4**	Negative regulation of MHC class II biosynthetic process
**5**	Regulation of cytolysis
**6**	Chemokine activity
**7**	Regulation of macrophage derived foam cell differentiation
**8**	Positive regulation of cAMP-mediated signaling
**9**	MHC class II biosynthetic process
**10**	Regulation of MHC class II biosynthetic process
**11**	Cxcr chemokine receptor binding
**12**	Macrophage derived foam cell differentiation
**13**	Foam cell differentiation
**14**	Chemokine-mediated signaling pathway
**15**	Negative regulation of megakaryocyte differentiation
**16**	Negative regulation of hematopoietic progenitor cell differentiation
**17**	Regulation of cAMP-mediated signaling
**18**	Negative regulation of signal transduction in absence of ligand
**19**	Negative regulation of extrinsic apoptotic signaling pathway in absence of ligand
**20**	Positive regulation of cAMP metabolic process

Biology Open • Supplementary information

Table S2. Proteins down-regulated in S1P$^{cko\text{-}Osx}$ BMCs. Table shows the top 15 proteins downregulated in S1P$^{cko\text{-}Osx}$ (Cko) BMC protein lysates by LC/MS/MS analysis, when compared to the WT (S1P$^{f/f}$).

	Gene	Protein	Protein Accession	*P*-val	FC (Cko/WT)
**1**	*Pf4*	Pf4 platelet factor 4	NP_064316	0.0000065	-1.99
**2**	*Rpl36a*	Rpl36a ribosomal protein L36A	NP_063918	0.13	-1.84
**3**	*Serpina3k*	Serpina3k serine (or cysteine) peptidase inhibitor	NP_035588	0.0014	-1.71
**4**	*Rin2*	Rin2 Ras and Rab interactor 2	NP_083000	0.17	-1.65
**5**	*Olfr694*	Olfr694 olfactory receptor 694	NP_666663	0.048	-1.64
**6**	*Myl4*	Myl4 myosin	NP_034988	0.013	-1.62
**7**	*Rps25*	Rps25 ribosomal protein S25	NP_077228	0.076	-1.59
**8**	*Med18*	Med18 mediator of RNA polymerase II transcription	NP_080315	0.0027	-1.58
**9**	*Serpinh1*	Serpinh1 serine (or cysteine) peptidase inhibitor	NP_033955	0.0034	-1.58
**10**	*Pafah1b3*	Pafah1b3 platelet-activating factor acetylhydrolase	NP_032802	0.0047	-1.58
**11**	*Fcrla*	Fcrla Fc receptor-like A	NP_660123	0.091	-1.54
**12**	*Rpl24*	Rpl24 ribosomal protein L24	NP_077180	0.022	-1.54
**13**	*Coq5*	Coq5 coenzyme Q5 homolog	NP_080780	0.022	-1.53
**14**	*Ppbp*	Ppbp pro-platelet basic protein	NP_076274	0.054	-1.5
**15**	*Rpl31*	Rpl31 ribosomal protein L31	NP_444487	0.23	-1.5

Table S3. Non-leukocyte clusters from WT and Cko from Sc-RNA-Seq analysis of WT and Cko BMCs using the 10x Genomics. Table shows the top 26 upregulated genes in each cluster, arranged in a descending order based on linear fold change (FC) values, plus two additional upregulated genes for most clusters, to establish maximum identity between WT and Cko. * identifies clusters that do not have an equivalent cluster in the opposite genotype. *P*-value, for every gene in every cluster, is extremely significant, markedly less than 0.05, almost equal to zero. Clusters have been ordered such that clusters with similar expression profiles and/or lineages are grouped together (rather than sequentially) and color-coded to identify an equivalent cluster in the opposite genotype, and an equivalent gene in the identical cluster. The absence of any color indicates lack of an equivalent cluster, or that a particular gene was not identified in the corresponding cluster, of the opposite genotype. Cko clusters are arranged such that their position in the table reflects an equivalent cluster in the WT. Suspected cell cluster lineages were determined using literature search of gene names and the use of CompBio (gtac-compbio.wsutl.edu), choosing the most likely theme that identifies the cell cluster depending on its positive gene expression profile and the expression of marker genes. Only genes with significant *P*-values and an expression fold ≥ 1.5 were used for analysis (often equaling approximately 100 genes or more). Roman numerals (I-IV) indicate different clusters that possibly have the same lineage, but still form a different cluster possibly due to a differentiation continuum. Clusters 0 and 2 in the WT, even though dissimilar within the first 26 genes, both displayed erythropoiesis as a strong theme when analyzed with a larger gene set, presumably at different maturation stages during erythropoiesis. Likewise for clusters 3, 5, 6, and 8 in the WT (corresponding to clusters 1, 8 and 6 in the Cko, respectively, with no corresponding cluster in the Cko for WT cluster 8) with these clusters likely representing different maturations states (differentiation continuum) in B cell lymphopoiesis. Cko Cluster 6 resembles WT cluster 6 only partially, but is classified in the B cell lineage due to strong Ly6d (marker gene) expression (seen in the Cko) (Jensen et al., 2016).

WT Clusters							Cko Clusters				
**Cluster 0**	**Cluster 2***	**Cluster 3**	**Cluster 5**	**Cluster 6**	**Cluster 8***	**Cluster 9***	**Cluster 4**	**Cluster 1**	**Cluster 8**	**Cluster 6**	**Cluster 9***
Pre-RBC-I	Pre-RBC-II	B cell-I	B cell-II	APC/B cell	B cell-IV	Macrophage	Pre-RBC-I	B cell-I	B cell-II	APC/B cell	T-cells/NK
Hbb-bs	*Car1*	*Vpreb3*	*Vpreb1*	*Ifitm1*	*Igkc*	*C1qc*	*Hba-a1*	*Vpreb3*	*Igll1*	*Ifitm1*	*Ccl5 (Rantis)*
Hbb-bs	*Tmem14c*	*Myl4*	*Igll1*	*Ctla2a*	*Ighm*	*C1qa*	*Hbb-bs*	*Pafah1b3*	*Vpreb1*	*Bst2*	*Ms4a4b*
Hba-a2	*C1qtnf12*	*Pafah1b3*	*Dntt*	*Bst2*	*Iglc2*	*C1qb*	*Hbb-bs*	*Ebf1*	*Dntt*	*Ly6d*	*AW112010*
Hba-a1	*Hebp1*	*Ebf1*	*Ebf1*	*Irf8*	*Cd74*	*Vcam1*	*Hbb-bt*	*Myl4*	*Ebf1*	*Cd74*	*Gzma*
Hbb-bt	*Smim1*	*Cd79b*	*Mif*	*Lgals1*	*Ly6d*	*Apoe*	*Hba-a2*	*Chchd10*	*Mif*	*Irf8*	*Nkg7*
Alas2	*Gstm5*	*Cd79a*	*Crip1*	*Ctsl*	*Ms4a1*	*Hmox1*	*Car2*	*Fcrla*	*Bfsp2*	*Cd7*	*Trbc2*
Gypa	*Car2*	*Pgls*	*Cd79a*	*Igfbp4*	*Hba-a2*	*Selenop*	*Gypa*	*Cd79a*	*Mzb1*	*H2-Aa*	*Cd3d*
Hemgn	*Fth1*	*Fcrla*	*Mzb1*	*Apoe*	*Hbb-bs*	*Fcna*	*Prdx2*	*Cd79b*	*Chchd10*	*Lgals1*	*Cd3g*
Rhd	*Glrx5*	*Ptprcap*	*Bfsp2*	*Tsc22d1*	*Hbb-bs*	*Axl*	*Alas2*	*Mzb1*	*Cd79a*	*H2-Ab1*	*Cd8b1*
Car2	*Rps2*	*Mzb1*	*Vpreb3*	*H2afy*	*Ifi30*	*Ctsb*	*Blvrb*	*Ptprcap*	*Crip1*	*H2-Eb1*	*Gimap4*
Mgst3	*Blvrb*	*Chchd10*	*Chchd10*	*Selenop*	*Hbb-bt*	*Lpl*	*Hmbs*	*Pgls*	*Vpreb3*	*Tcf4*	*Klrd1*
Prdx2	*Minpp1*	*Lrmp*	*Id3*	*Ccnd2*	*Fcmr*	*Sdc3*	*Rhd*	*Igkc*	*Blnk*	*Tsc22d1*	*Ctla2a*
Slc4a1	*Acp5*	*Cnp*	*Npm1*	*Egfl7*	*Hba-a1*	*Psap*	*Mgst3*	*Tifa*	*Tspan13*	*Gas5*	*Skap1*
Hmbs	*Prdx2*	*H2afv*	*Ptprcap*	*Tmem176b*	*Snca*	*Marcks*	*Hemgn*	*Cnp*	*H2afy*	*Rpl31*	*Ltb*
Snca	*Cldn13*	*Tifa*	*Lef1*	*Tcf4*	*Cd79a*	*Ctsd*	*Alad*	*Herpud1*	*Id3*	*Ramp1*	*Shisa5*
Ctse	*Klf1*	*Lgals9*	*Ncl*	*Ramp1*	*Cd79b*	*Slc40a1*	*Glrx5*	*H2afx*	*Vpreb2*	*Rnase6*	*Lck*
Alad	*Metap2*	*Blnk*	*Lrmp*	*Gas5*	*Alas2*	*Aif1*	*Cpox*	*H2afv*	*Eef1g*	*Hspe1*	*Thy1*
Slc25a37	*Mgst3*	*Pou2af1*	*Lgals9*	*AW112010*	*Tnfrsf13c*	*Pld3*	*Slc4a1*	*Tubb5*	*Npm1*	*Srgn*	*Cd2*
Blvrb	*Aqp1*	*Siglecg*	*H2afy*	*Plac8*	*Rsad2*	*Maf*	*Ctse*	*Ptma*	*Hmgn1*	*Hsp90ab1*	*Ctsw*
Hagh	*Cox6b2*	*Bach2*	*Fkbp1a*	*Fxyd5*	*H2-Aa*	*Trf*	*Ubac1*	*Elof1*	*Hes1*	*Rps11*	*Gimap6*
Odc1	*Bola3*	*Zfp706*	*Hsp90ab1*	*Mif*	*H2-Eb1*	*Cfp*	*Klf1*	*Dnajc7*	*Ptp4a3*	*Lat2*	*Bcl2*
Ubac1	*Ctse*	*Smarca4*	*Ptma*	*Nkg7*	*Malat1*	*Grn*	*Urod*	*Pou2af1*	*Ptprcap*	*Gata2*	*Septin-1*
Fech	*Atpif1*	*Ptma*	*Phgdh*	*Srgn*	*Bpgm*	*Sirpa*	*Odc1*	*Tcf3*	*Phgdh*	*Bmyc*	*Cd3e*
Gch1	*Rplp1*	*Dnajc7*	*Hspe1*	*Calr*	*Gch1*	*Mrc1*	*Cldn13*	*Smarca4*	*Ncl*	*Npm1*	*Lat*
Cpox	*Eef1g*	*Cd72*	*Hmgn1*	*Shisa5*	*Mkrn1*	*AW112010*	*Snca*	*Cd72*	*Bin1*	*Pepb1*	*Hcst*
Urod	*Bex3*	*Arl5c*	*Tspan13*	*Pebp1*	*Ltb*	*Lgmn*	*Atpif1*	*Hmgb1*	*Lef1*	*Bcl11a*	*H2-Q7*
Klf1		*Herpud1*	*Blnk*	*Rpl31*		*Cd68*	*Slc25a37*	*Blnk*	*Fkbp1a*	*H2afy*	
Glrx5		*Tubb5*	*Eef1g*	*Rnase6*			*Hagh*	*Siglecg*	*Hsp90ab1*	*Ccnd2*	

Biology Open • Supplementary information

SUPPLEMENTARY REFERENCES

Jensen, C.T., S. Lang, R. Somasundaram, S. Soneji, and M. Sigvardsson. 2016. Identification of Stage-Specific Surface Markers in Early B Cell Development Provides Novel Tools for Identification of Progenitor Populations. *J Immunol*. 197:1937-1944.

Biology Open • Supplementary information
